# 3,4-Ethylenedioxythiophene (EDOT) End-Group Functionalized Poly-ε-caprolactone (PCL): Self-Assembly in Organic Solvents and Its Coincidentally Observed Peculiar Behavior in Thin Film and Protonated Media

**DOI:** 10.3390/polym13162720

**Published:** 2021-08-14

**Authors:** Anca-Dana Bendrea, Luminita Cianga, Gabriela-Liliana Ailiesei, Elena-Laura Ursu, Demet Göen Colak, Ioan Cianga

**Affiliations:** 1 Centre of Advanced Research in Bionanoconjugates and Biopolymers, “Petru Poni” Institute of Macromolecular Chemistry, 41A, Grigore-Ghica Voda Alley, 700487 Iasi, Romania; anca.bendrea@icmpp.ro (A.-D.B.); ursu.laura@icmpp.ro (E.-L.U.); 2 NMR Spectroscopy Department, “Petru Poni” Institute of Macromolecular Chemistry, 41A, Grigore-Ghica Voda Alley, 700487 Iasi, Romania; gdarvaru@icmpp.ro; 3 Department of Chemistry, Faculty of Science and Letters, Istanbul Technical University, Maslak, Istanbul 34469, Turkey; goende@itu.edu.tr

**Keywords:** poly-ε-caprolactone, 3,4-ethylenedioxythiophene (EDOT), self-assembly, electroactive macromonomers, amphiphiles, polymer single crystals, micelles, ^1^H- and 2D-correlated ^1^H-^1^H-NMR spectroscopy analysis, atom force microscopy (AFM)

## Abstract

End-group functionalization of homopolymers is a valuable way to produce high-fidelity nanostructured and functional soft materials when the structures obtained have the capacity for self-assembly (SA) encoded in their structural details. Herein, an end-functionalized PCL with a π-conjugated EDOT moiety, (**EDOT-PCL**), designed exclusively from hydrophobic domains, as a functional “hydrophobic amphiphile”, was synthesized in the bulk ROP of ε-caprolactone. The experimental results obtained by spectroscopic methods, including NMR, UV-vis, and fluorescence, using DLS and by AFM, confirm that in solvents with extremely different polarities (chloroform and acetonitrile), **EDOT-PCL** presents an interaction- and structure-based bias, which is strong and selective enough to exert control over supramolecular packing, both in dispersions and in the film state. This leads to the diversity of SA structures, including spheroidal, straight, and helical rods, as well as orthorhombic single crystals, with solvent-dependent shapes and sizes, confirming that **EDOT-PCL** behaves as a “block-molecule”. According to the results from AFM imaging, an unexpected transformation of micelle-type nanostructures into single 2D lamellar crystals, through breakout crystallization, took place by simple acetonitrile evaporation during the formation of the film on the mica support at room temperature. Moreover, **EDOT-PCL’s** propensity for spontaneous oxidant-free oligomerization in acidic media was proposed as a presumptive answer for the unexpected appearance of blue color during its dissolution in CDCl_3_ at a high concentration. FT-IR, UV-vis, and fluorescence techniques were used to support this claim. Besides being intriguing and unforeseen, the experimental findings concerning **EDOT-PCL** have raised new and interesting questions that deserve to be addressed in future research.

## 1. Introduction

Today, a plausible way to find new functions of conventional polymers, in order to better fit a given application, is through high-precision polymers with complex architectures [1]. As different properties have been demonstrated through a comparison with the classical, linear arrangement of the chain [2], the goal of producing polymers with complex architectures can be achieved by applying the concept “from functionality to function”. In this regard, polymeric materials with specific chain-end functionality have become accessible, especially through “living” controlled polymerizations [3,4], which have led to the production of valuable materials with new and improved properties [5,6,7] and to reactive intermediates of great interest for the construction of polymers with defined molecular architectures [4,8,9]. In bulk, in solution, or in film, placed at the chain-end of the homopolymers or copolymers, and even in blends of them, the end-groups impact their properties at both the molecular and supramolecular level. It is interesting that, in spite of the small content of terminal groups as compared to the macromolecular backbone, this effect is more pronounced if they are placed in confined spaces. One consequence of the confinement effect caused by the end-group is that the block copolymers morphology is radically modified, resulting in unparalleled property changes [8]. Additionally, the existing polymer properties, such as enzymatic degradation [6], of the solution–protein interaction [10] or the drug loading capacity [5] can be manipulated through the functional end-groups, or other new properties can be added such as fluorescence properties [7,11].

Basically, there are two approaches to the synthesis of end-functional polymers: (i) α-functionalization or the so-called “initiation method”, which makes use of a functionalized initiator in a controlled type polymerization and (ii) ω-functionalization, which can be accomplished post-polymerization, by modifying the existing chain ends with the desired functionality; a combination of these variants can be applied.

Various types of functional end-groups have been introduced into a plethora, and progressively, a diverse range of polymers, such as (poly(ethyleneglycol) (PEG) [12], poly(lactide) PLA [13], poly(N-alkyl-oxazoline)s (POXA) [14], poly(N-isopropylacrylamide) (PNIPAM), poly(methyl methacrylate) (PMMA), poly(ethylene glycol methyl ether methacrylate) POEGMA [15], polystyrene (PSt) [16,17], polyethylene, (PE) [18], and polypeptides [19], using various ”living” polymerization methods, including atom transfer radical polymerization (ATRP) [16], nitroxide-mediated polymerization (NMP) [17], reversible addition–fragmentation chain transfer (RAFT) [15], and ring-opening polymerization (ROP [13,19], CROP [14], and ROMP [18]). These are often combined with post-polymerization chemical modifications [12,16]. Besides these polymers, PCL, which belongs to the first generation of synthetic aliphatic polyesters [20], is among the earliest studied biomaterials of synthetic nature. The biodegradability of PCL has led to its extensive exploration for various bioapplications [21].

Being widely commercially available, PCL has a high capacity to be miscibly blended, and this has resulted in interesting compatibilization studies [22,23,24]. Whether in blends or copolymers of various architectures [25,26,27], PCL is easily shaped and manufactured in the form of hydrogels [22,23], nanoparticles [24], nanofibers [27,28], and, more importantly, due to its crystallizable nature, into a well-defined and geometrically shaped 2D lamellar single-crystal structure that has various and interesting applications [25,29].

Due to the renewed interest in ε-caprolactone-derived components for the construction of systems with advanced functions, various functional groups have been added by the end-group functionalization. Thus, innovative uses of ε-caprolactone-based segments in sophisticated polymer architectures or micellar systems have also been noted [21]. Thus, the end-group functionalization of PCL means that it can be applied in, for example, more efficient drug delivery systems [5,11] and in aggregation-induced emission (AIE) materials [7], as catalytically active recyclable materials, or as magnetically responsive, luminescent materials [29]. Moreover, functional groups have often been added to PCL to transform it into a reactive intermediate in order to subsequently use them in coupling reactions. These reactions led to various macromolecular structures [26,30], macroinitiators [31,32], and, very importantly, well-defined macromonomers [33,34,35]. Macromonomers are, in fact, oligomer or polymer chains that are functionalized with polymerizable end-groups. The homopolymerization of macromonomers, which results in “polymacromonomers”, has evolved as a strong strategy to obtain “(macro)molecular (bottle)brushes” or “cylindrical polymer brushes” [36,37]. Today, this class of complex polymer architectures works as a versatile toolbox of anisotropic building blocks for next-generation nanomaterials [37]. These are suitable for applications in key fields, including nanomedicine [38].

Electroactive macromonomers, so named due to their ability to oxidatively polymerize using electrochemical techniques [12,17], are also active in chemically oxidative polycondensation [13,14,15,16] or, alternatively, in other polymerization methods specifically designed to obtain conjugated polymers (CPs) [9,34]. These macromonomers are important building blocks, the use of which has enabled the enlargement of the CPs “hairy-rods” class [39] and has allowed for well-defined oligomeric/polymeric flexible side chains to be attached to the conjugated, rigid backbones, resulting in graft ***rod-coil*** type polymers with a self-assembling propensity and high flexibility in the tuning of properties. Several such PCL-derived electroactive macromonomers have already been reported [40,41,42].

In some cases [41,42], the introduction of PCL side chains to the conjugated backbones has contributed to increased biocompatibility and efficiency when working in biorelevant aqueous electrolytes of the obtained materials. In the case of a heterografted polythiophene, such introduction contributed to the development of a material whose weight was composed mainly of PCL and PEG commodity polymers [43], which also showed that its amphiphilic nature allows for the manipulation of the self-assembled morphology by solvent selectivity and concentration of solutions.

Therefore, it is clear that in being endowed with the unique combination of polymer and monomer-like properties, the macromonomers’ structural details are reflected in the characteristics of the materials that are constructed based on them. Thus, as electroactive macromonomers’ behavior in instances with significance for further applications, it is a rational, necessary step; in this paper, we focused on properties in solution and on the morphology in thin films of such a compound.

**Scheme 1:**
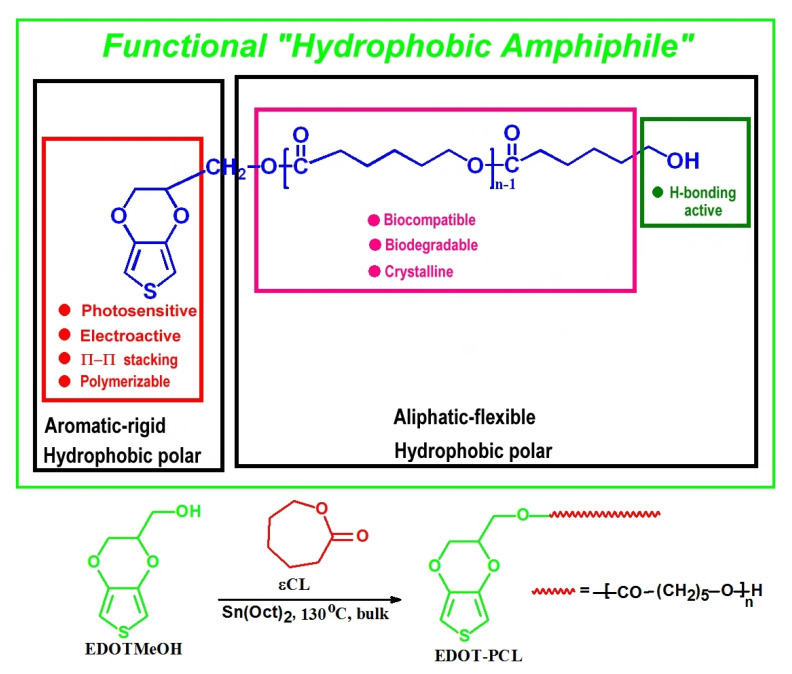
The functional characteristics of the EDOT-PCL macromonomer and the pathway for its synthesis.

Based on our previous experience with electroactive macromonomers synthesis [12,14,16,17], we recently engineered and reported [42] **EDOT-PCL** (Scheme 1) as a new structure, based on EDOT and oligo-ε-caprolactone (OCL). Our option was made with the knowledge that OCL, which has a molecular weight of less than 3000 Da, undergoes phagocytosis by the phagosomes of macrophages and giant cells, followed by intracellular degradation by esterases. This type of OCL in various combinations can produce useful biomaterials that solve the slow degradation problems of PCL [44].

The reported macromonomer, **EDOT-PCL**, was shown to be the key element in the incorporation of CP PEDOT into a 3D polymeric flexible and electroactive bioplatform. It was also shown to have favorable properties that enhanced the tissue integration performance of implantable medical devices [42].

It is noteworthy that, very recently, a combination of PEDOT or oligomers of EDOT with PCLs, as blends [45] or as linear and hyper-branched copolymers [46] in the form of nanofibers or hydrogels, appropriate for skeletal muscle or neural tissue engineering, was also developed. This emphasizes the current interest in the combination of the two for bioapplications.

Scheme 1 shows the reaction path of **EDOT-PCL** synthesis and, in a schematic manner, the design criteria that were taken into account in tailoring it. OCL, in addition to being biocompatible, with a high ionic conductivity, ion solvation capacity, and a wide window of electrochemical stability [42], is a well-known hydrophobic, flexible, semicrystalline, polar, and biodegradable polymer [21]. In contrast, EDOT is a flat, aromatic, and rigid combination of two fused rings. Its electroactivity and polymerization capacity have recently been demonstrated [42].

Moreover, due to its aromatic character, this fragment is photosensitive and is capable of non-covalent intermolecular stacking through π–π interactions. At the opposite end, **EDOT-PCL** exposes a hydroxyl group, which could not only be used for subsequent chemical modifications but could also form intermolecular hydrogen bonds.

Due to the aforementioned differences in the structural details of its components, we associated **EDOT-PCL** with the term “hydrophobic amphiphile” due to its design being similar to π-conjugated fullerene-C60-alkyl molecules introduced by Nakanishi et al. [47]. These authors extended the amphiphilic assembly concept in aqueous environments to molecules that are solely made up of hydrophobic domains. In organic solvents, self-assembled structures, similar to the structures of classical amphiphiles, were demonstrated [47].

Moreover, an experimental exploration unexpectedly revealed that microphase separation behavior is sometimes witnessed in a single polymer during end-group functionalization [48,49]. In most cases, this occurs in polymers with flexible backbones and bulky terminal groups that are capable of strong planar interactions [8].

However, anisotropy in shape and stiffness has only recently been recognized as an important parameter in the fine-tuning of the self-assembled structures of the so-called “shape amphiphiles”. In addition to the difference in interactions with solvents, which is inherent to some degree in any block system, the shape effect and the mismatch in rigidity are overwhelming factors that lead to the self-assembly of compounds that are constructed based on the characteristic criteria of the “shape amphiphiles” [50].

As can be seen in Scheme 1, the **EDOT-PCL** structure shows almost all the “ingredients” mentioned above for particular behaviors. Thus, in this study, we focused on showing the impact of the final EDOT group on OCL properties and investigating the **EDOT-PCL** capacity of self-assembly in solution and the subsequent morphological transitions that occurred during thin film formation. The solvents used, chloroform (Chl) and acetonitrile (ACN), were deliberately chosen in order to show the significant influence caused by their difference in polarity and because they are commonly used as reaction media for chemical and electrochemical polymerization [42,43,51]. An explanation is also given for the transition from colorless to blue color that was observed during the preparation of a concentrated solution of **EDOT-PCL** in CDCl3.

## 2. Materials and Methods

### 2.1. Materials

Hydroxymethyl-3,4-ethylenedioxythiophene (EDOTMeOH; 95% Sigma-Aldrich, Darmstadt, Germany), ε-caprolactone (ε-CL Aldrich, Darmstadt, Germany), and stannous octanoate (Sn(Oct)_2_) (Sigma, Darmstadt, Germany) were used as received. All the solvents were purified and dried using the usual methods.

### 2.2. Synthesis of EDOT-PCL Macromonomer

Under nitrogen, 21.75 mmol of ε-CL, 1.45 mmol of EDOTMeOH and 0.00725 mmol of Sn(Oct), in a ratio of (I)/(M)/(cat) = 200/3000/1 were added to a previously flamed and nitrogen-purged two-neck round-bottom flask that was equipped with a dropping funnel and a magnetic stirrer. The ε-CL polymerization was carried out in bulk at 130 °C. After 24 h of reaction, the resulting mixture was diluted with an amount of CHCl_3_ and was poured into a 10-fold excess of cold methanol. The resulting **EDOT-PCL** macromonomer was collected after filtration and was dried at room temperature in a vacuum for 3 days [42].

### 2.3. Measurements

The NMR spectra were recorded on a Bruker Avance NEO 400 MHz spectrometer (Bruker BioSpin GmbH, Rheinstetten, Germany, ) operating at 400.1 MHz for ^1^H and 100.6 MHz for ^13^C nuclei, equipped with a 5 mm four-nuclei direct detection z-gradient probe using standard pulse sequences, as delivered by Bruker with TopSpin 4.0.8 spectrometer control and processing software. Chemical shifts are reported in δ units (ppm) and were referenced to the residual solvent signal (CD_3_CN at 1.93 ppm and 118.19 ppm for ^1^H-NMR and ^13^C-NMR, respectively; CDCl_3_ at 7.26 ppm for ^1^H-NMR). All the experiments were recorded at room temperature (24 °C). The 2D ^1^H-^1^H NOESY spectrum was recorded using a phase-sensitive NOESY pulse sequence, with a number of scans of 64, a spectral resolution of 2.37 Hz, and a mixing time of 0.8 s.

The FTIR spectra were recorded on a Bruker Vertex 70 FTIR spectrometer in the transmission mode using KBr pellets.

The molecular weight of the **EDOT–PCL** macromonomer was estimated from ^1^H-NMR data, using a similar algorithm as previously reported [42], and the obtained value of M_n_^1^_H-NMR_ (1998 Da) indicated a degree of polymerization (PD) of 16. Thus, regardless of the solvent used for the NMR registration, it can be seen that the obtained result was in line with the previously reported result (PD = 16.5) obtained for CDCl_3_ [42].

UV-vis absorption spectra were measured using a Specord 200 Analytik Jena spectrophotometer. Fluorescence measurements were carried out using a Perkin Elmer LS 55 apparatus. The measurements were performed in CHCl_3_ and acetonitrile with the concentrations of the solutions kept constant at 1 mg/mL (0.49 × 10^−6^ M). The same concentration was used for measurements of EDOTMeOH.

Particles characterization was carried out using dynamic light scattering (DLS). A Malvern Zetasizer Nano ZS instrument equipped with a 4.0 mW He–Ne laser, operating at 633 nm, at a detection angle of 173°, was used. The intensity-weighted mean hydrodynamic size (Z average) and the polydispersity factor were obtained from an analysis of the autocorrelation function. Samples were used as prepared, without filtration at a concentration of c = 1mg/mL (0.49 × 10^−6^ M) in CHCl_3_ and acetonitrile. The reported values represent the average of the three measurements of each sample, at 25 °C, with an equilibration time of 5 min before each measurement. Quartz cells were used for all the measurements.

The morphology of the **EDOT-PCL** thin film was highlighted by atomic force microscopy (AFM) using an NTEGRA Spectra (NT-MDT, Russia) instrument with commercially available silicon nitride cantilevers (NSG10, NT-MDT, Russia). The samples were obtained by drop-casting the **EDOT-PCL** solution in the given solvent at a concentration of 1 mg/mL (0.49 × 10^−6^ M) on freshly cleaved muscovite mica and were dried at ambient temperature before observation. To allow for uniform evaporation, the samples were kept in a solvent-saturated atmosphere during film formation by using a glass dome that only partially covered them. The resulting topographical AFM images were analyzed using Nova 1.0.26.1443 software.

## 3. Results and Discussion

### 3.1. Behavior of EDOT-PCL in Solution

In order to investigate the properties of **EDOT-PCL** in solution, and more specifically, to investigate its propensity for SA in solvents with different selectivities and the spontaneous formation of self-assembled structures by direct dissolution, some appropriate techniques were used. Thus, nuclear magnetic resonance spectroscopy (NMR), UV-vis and fluorescence spectroscopy, as well as DLS were chosen as methods sensitive not only to the intrinsic solvent effects but also to those that occur as a result of a change in the solute conformation or structure due to the change in the solvent. Even more specifically, the NMR technique is suitable because it has been extensively used to study the organization and structure of molecular brushes in solution [52], in order to establish polymers’ microstructure [53] and to determine polymers’ absolute molecular weight [54] and their regioregularity [55]. Solvent-dependent ^1^H-NMR studies, conducted to prove the formation of micelles from amphiphilic structures [55,56], were also performed.

**Figure 1:**
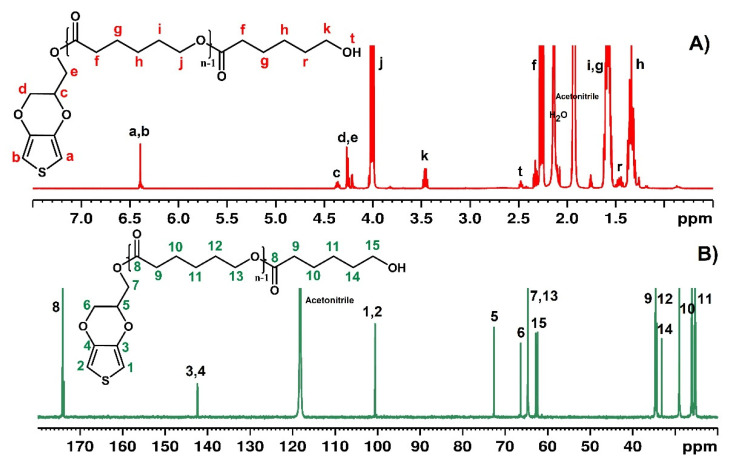
^1^H- (**A**) and ^13^C- (**B**) NMR spectra of **EDOT-PCL** macromonomer in CD_3_CN.

The ^1^H-NMR and ^13^C-NMR spectra, corresponding to the **EDOT-PCL** macromonomer, which were registered in CD_3_CN, and their assignment of proton and carbon signals according to structural formulas, are presented in Figure 1. A detailed discussion of them can be found in Supplementary Materials (SM). By comparing these spectra with previously published data, for which the **EDOT-PCL** macromonomer NMR registration was performed in deuterated chloroform (CDCl_3_) [42], an increased polarity of CD_3_CN was shown to influence the position of the signals in the ^1^H-NMR spectrum, as was expected [57]. Thus, most of the proton signals were upfield-shifted in CD_3_CN, as can be seen by comparing to the values registered in CDCl_3_ (see Figure S1 and Table S1 in SM). Following this comparison, it can be seen that the position of the signal that corresponds to the CH_2_ group (denoted **k**), which is directly bonded to the hydroxyl-end of the OCL chains, was most affected (Δδ = 0.22 ppm). This is an expected phenomenon because acetonitrile, in spite of it being a weaker hydrogen-bonding acceptor [58], has a higher polar character in comparison to chloroform and shows a more pronounced solvation ability to the hydroxyl moiety (–OH) situated into the neighborhood of protons **k**. Moreover, hydrogen bonds of ACN with the –OH groups in the OCL chain ends were not excluded; these types of bonds have already been reported for phenols and ethanol [59,60]. Thus, this environmental modification around protons **k**, when compared to CDCl_3_ registration, results in an increased density of the electrons, which could be the reason for the registered shifting. Another important difference, as shown in Figure S1, is the discernible downfield-shifting in the range 6.36–6.38 ppm, which is was assigned to the aromatic **a** and **b** protons of the thiophene ring. The explanation for this opposite influence of the CD_3_CN on the two different constitutive parts of **EDOT-PCL** could be found in its specific interaction with the aromatic thiophene ring in EDOT moiety, which significantly differs from the interaction with aliphatic CH_2_-methylene in OCL repeating units. Thus, if the values of the dipole moments of the CDCl_3_, CD_3_CN, and EDOT ring are considered (see Table S2 in SM), it can be assumed that the interaction of CD_3_CN molecules with the EDOT moiety is favored compared to CDCl_3_ molecules. This is due to the higher polar character of CD_3_CN, which results in a stronger dipole–dipole interaction with the fused rings of EDOT. If the solute-solvent interaction is taking place more preferentially in the region of the ethylenedioxy ring of EDOT, then it results in the deshielding of the thiophene ring of EDOT, which can explain the modification that was observed in the NMR spectrum of protons **a** and **b** (Figure S1A).

Moreover, if the general principle that an increase in solvent polarity leads to a deshielding effect in the fully soluble parts, which results in downfield-shifting, is taken into account, the NMR investigations confirmed the known character of ACN as a poor/non-solvent for PCLs [61,62]. Meanwhile, the upfield-shifting of **a** and **b** aromatic protons in Chl is striking evidence of the **EDOT-PCL** intermolecular π–π stacking of the aromatic segment of the molecule [63] in this solvent, thus suggesting the formation of associated supramolecular structures.

This opposite “upfield–downfield” shifting, observed in a solvent-dependent NMR study of **EDOT-PCL**, emphasizes a net difference in the interaction of the solvents used with the aromatic, rigid, and planar parts on the one hand and the aliphatic, flexible OCLs on the other hand. It also leads to the idea that, at the supramolecular level, the aromatic and aliphatic parts of **EDOT-PCL** could be placed in spatially different regions in the solution. As NMR is one of only a few techniques that allow for the determination of the molecular spatial location, due to their intra- and inter-molecular interactions, we made use of the two-dimensional nuclear Overhauser effect spectroscopy (2D ^1^H-^1^H-NMR NOESY) variant in order to evaluate the ACN-induced behavior of **EDOT-PCL**.

Based on the through-space dipole–dipole interaction, NOESY experiments have provided significant information on both the intra- and inter-molecular proximity of protons [64] that have sufficient rotational mobility [65] and which had internuclear distances that were typically <0.5 nm. This provided excellent insights into the nature of the micelle-forming process [65,66,67], which is applicable in any kind of self-assembled structure, for any geometry, and for any type of constituents [65].

**Figure 2:**
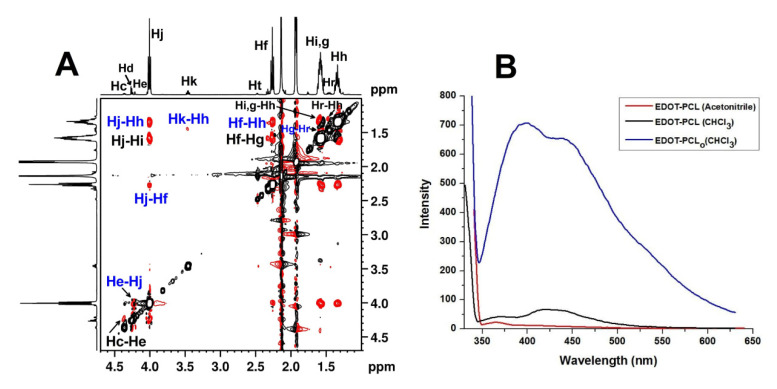
(**A**) 2D NOESY ^1^H–^1^H spectrum of **EDOT-PCL** in CD_3_CN (0–4.7ppm range). (**B**) Fluorescence spectra of **EDOT-PCL** macromonomer and its oligomerized form (**EDOT-PCL_o_**) in the used solvents at concentrations of 1 mg/mL, (0.49 × 10^−6^ M), and λ_excit_ = λ_max_abs (see Table S3).

In Figure 2A, which represents the chemical shift region between 1.0 and 4.7 ppm from the 2D NOESY ^1^H-^1^H spectrum of **EDOT-PCL** in CD_3_CN (see Figure S2 for the whole investigated range), the identified through-space couplings are marked by blue letters in order to differentiate them from the COSY cross-peaks between the *J*-coupled protons (noted with black letters), which also appear in the NOESY spectrum. The existence of cross-peaks confirms that magnetization transfer is possible and that there is proximity between the different protons, even transient ones. Moreover, as these peaks are negative, as previously reported [68], they are evidence of fast motion in the solution of the flexible part of **EDOT-PCL** molecules. It has also been stated that the cross-peaks that are assigned to intramolecular interactions are naturally more pronounced than those for intermolecular correlations [65]. In Figure 2A, peaks attributed to H**_j_**–H**_h_** and H**_f_**–H**_h_** interactions have the highest intensities, and they characterize each OCL-repeating unit’s intramolecular behavior. Their appearance is expected so long as the length of one of the PCL-repeating units, calculated from the crystallographic data, is 0.86 nm [69]; thus, the distance between the **j** and **h** or **f** and **h** protons will most likely be in the range ≤0.5 nm. With a lower intensity, cross-peaks are also present in Figure 2A and are due to the interactions (intra- but also intermolecular ones) between protons **g** and **i** and protons **j** and **f**. In the case of intramolecular interactions, the presence of the peak H**_j_**–H**_f_** is important because it sustains the possibility of there being a more collapsed OCL chain in a marginal ACN solvent, thus allowing **j** and **f** protons to be placed at a distance of around 0.5 nm. The presence of the peak H**_e_**–H**_j_** is important for sustaining our claim of **EDOT-PCL** SA, and thus for the presence of EDOT and OCL in different spatial areas.

This is the only peak that is specific to the interaction between a proton that is reminiscent of the initiator and a proton that belongs to the OCL chain. Its existence proves the fact that this flexible part from the EDOTMeOH initiator shares the same space/area with the OCL chains, e.g., the core of a micelle-like particle or a borderline space between the core and the shell of these supposedly formed micellar particles. Moreover, there are presently no peaks characterizing interactions between proton **e** and any of the nearest **c** or **d** protons in the EDOT moiety. This is also evidence for a different placement in the space of the aromatic heterocycle.

In completion of the NMR data, photophysical properties of **EDOT-PCL** in the same solvents were investigated, as well. It is known that such analyses mirror the self-assembly phenomena in solution [70,71,72,73]. Figure S3A presents the traces of the absorption spectra of **EDOT-PCL** in the two investigated solvents at a concentration of 0.49 × 10^−6^ M. This concentration was maintained the same for all the other investigations (DLS and AFM). The traces in Figure S3A show a vibronic aspect and a high absorbance. It was demonstrated that these are due to a high value of the solution concentration. When the registration was performed at a concentration of 0.245 × 10^−6^ M, the appearance of the curves becomes smooth (Figure S3B) and without peaks position modification in comparison to those registered at the higher concentration. In the two investigated solvents, λ_max_abs of the peak with the highest absorbance appears in almost the same position (254 nm in ACN and 257 nm in Chl).

The bimodal curve in ACN has a similar shape as a reported TEMPO-containing EDOT compound (also obtained from EDOTMeOH), which shows λ_max_abs at 256 nm [74] and of bare EDOT, registered in ethanol [75], with the two absorption peaks at 215 nm and 243 nm, respectively.

Moreover, in Figure S3C, the UV-vis absorption traces of the EDOTMeOH, used as an initiator in the synthesis of **EDOT-PCL**, and registered in the same solvents, presented shapes that were similar to those of **EDOT-PCL**, with λ_max_abs values (Table S3) being practically unchanged for ACN and with only a 5 nm red-shift for Chl.

The conclusion can be drawn that the introduction, through an ester linkage, of the OCL chain at the third position of the EDOT fused rings had practically no influence on its UV-vis absorption properties, and as expected, that EDOT moiety was the only absorbing/emitting (Figure 2B) component of **EDOT-PCL**. A slight shifting of the λ_max_abs, of about 3 nm, was observed in the traces of the two solvents for **EDOT-PCL** (Figure S3A), and this confirms the previous claim that the absorption phenomenon is insensitive to the solvents’ polarity change and only affects the excited state [76]. Figure 2B shows the changes in the photoluminescence behavior of **EDOT-PCL**, which can be correlated with the change in the solvents’ dielectric constant. Thus, the two traces corresponding to the Chl and ACN solutions, at identical concentrations, which are non-symmetrical, bimodal, and broad, cover a range of approximately 200 nm and have emission maxima (λ_max_em) placed between the violet-blue and blue regions (365–430nm). A λ_max_em hypsochromic shift of 5 nm for the first emission maximum and 35 nm for the second was observed when the solvent polarity increased from Chl to ACN (Table S3 in SM). This shift was accompanied by a decrease in the intensity of the fluorescence. In addition, the magnitude of the low-energy peak increased in Chl. However, it is also important to emphasize that the fluorescence traces of the EDOTMeOH initiator, which were registered in the same solvents, were monomodal but broad and non-symmetrical (Figure S3D), with a blue-shifted λ_max_em at 350 nm and 325 nm (see also Table S3) compared to **EDOT-PCL**. The possible explanations for all phenomena described above are as follows: (i) the blue-shifting of λ_max_em registered in the polar ACN solvent could be due to the stabilization of the ground state of **EDOT-PCL,** which has an increased polar character than the excited state; (ii) the bimodal shape and presence of the red-shifted peaks in both the investigated solvents, (the last one being a mark for the formation of the nanoparticles/micelle-like particles [55,72,77]), could be proof of the self-assembling nature of **EDOT-PCL** in the solution; (iii) the increased values of apparent hydrodynamic diameter (D_h_) of the self-assembled structures in ACN in comparison with those formed in Chl, experimentally determined (vide infra) could be the reason for the observed fluorescence intensity decrease in this solvent. This behavior followed the well-established phenomenon of the dependence of fluorescence intensity on particle size [7,55]. In general, the results obtained from the fluorescence measurements lead to the conclusion that particular interactions with each of the solvents used in OCL chains (as a major component of **EDOT-PCL**) as well as OCL-adopted conformation (extended in Chl or collapsed in ACN) are two of the most influential factors on this property. Additionally, specific EDOT planar geometry and peculiar inter-rings interaction besides their relative orientation, which is driven by the ring aromatic nature and by the S and O heteroatoms presence, can contribute to the noticed behavior [78,79]. In addition, the presence of OCL, as a substitute in the third position of the EDOT fused rings, has no negative impact on the λ_max_em values when compared to that of the starting, non-assembled EDOTMeOH. In a similar manner to what was previously reported for PEG [75,80], OCL from the shell of micellar particles works as an electronic shield to preserve and protect the fluorescence of EDOT in the core.

In addition to the NMR measurements and to the photophysical properties that only suggested the formation of the **EDOT-PCL** self-assembled structures, DLS can be used as a technique that “senses” the presence of such entities and more certainly probes their formation [7,25]. DLS traces, as intensity-weighted distribution of D_h_, shown in Figure S4A, are multimodal in both solvents (bimodal in Chl and trimodal in ACN), and this emphasizes the possible concomitant presence of colloidal particles of various sizes or clusters formed by them in solution. In both solvents, most of the formed structures (approximately 88%) had a D_h_ of 184 nm in Chl and 196 nm in ACN. The higher value of D_h_ in ACN could be due to the fact that PCL has a collapsed conformation. Consequently, a higher number of **EDOT-PCL** molecules, exposing the fused rings to ACN solvent, should assemble together in order to attain colloidal stability. The largest values of 4800 nm in Chl and 5500 nm in ACN could be indicative of the eventual presence of particle clusters. These high values can also be due to the presence of particles that do not have a spherical/globular shape because of the limitations of the DLS technique. In general, the information given by DLS measurements should be viewed with utmost care. Thus, an argument for the limitations of the DLS result, is also represented by the traces shown in Figure S4B. This figure shows the traces as volume-weighted distribution of apparent D_h_, which is bimodal for both solvents. For the solution in Chl, the apparent D_h_ has almost the same values as those found in intensity-weighted measurement. The only difference is referring to the volume contribution of the particles with higher D_h_, which is increased as compared to that obtained in the same solvent in Figure S4A. For the ACN solution, the peaks which in intensity-weighted representation appeared at higher values (196 and 5400 nm) are missing in trace in Figure S4B. This missing suggests that the volume contribution of these particles is also negligible. In both Figure S4A,B, the smallest values of the apparent D_h_, of only 4.3 nm and 3.13 nm, respectively, which were determined in ACN, could be due, hypothetically, to the presence of particles that are formed from only several associated molecules or due to unimolecular micelle-like aggregates. In order to roughly decipher this aspect, the theoretical diameter of gyration (D_g_) [81] of 2.28 nm was obtained, based on the value of the oligo-ε-caprolactone’s radius of gyration (R_g_) [82]. This value resulted from the calculations that are included in the SM. If the collapsed conformation of PCL in the marginal ACN solvent is accepted, the colloidal particles of only 4.3 and 3.13 nm would be considered to be micelle-like particles formed from more than two **EDOT-PCL** molecules.

### 3.2. Surface Morphology Analysis of EDOT-PCL Thin Films

**Figure 3:**
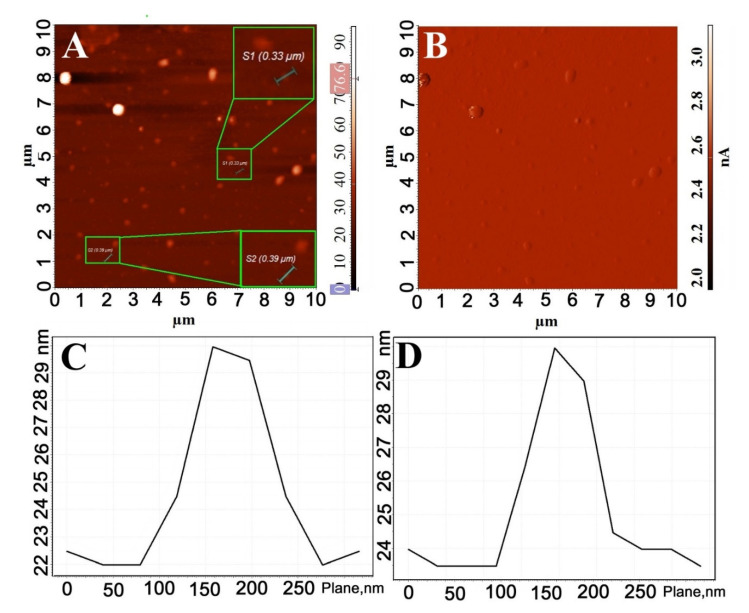
(**A**) AFM height image of the round-shaped particles formed by drop-casting an EDOT-PCL solution in Chl; (**B**) AFM amplitude image; (**C**,**D**) cross-sectional analysis of the particles shown in magnified insets in (**A**): (**C**) S1 section and (**D**) S2 section.

Different microscopy techniques are commonly used to visualize the morphology of solution-based films, even if there is a clear distinction between the self-assembled structures in the solution that could be affected by their transformations during solvent evaporation and film-forming. This is because such a process involves both the physical properties of the support used and the colloidal stability of the solution-formed particles. Thus, the presence of particles with variable sizes (with an average lateral width of around 200 nm) as a dense, uniformly scattered network, as shown in the AFM images in Figure 3, confirm that the self-assembled structures of **EDOT-PCL** in Chl, detected by DLS, are round in shape. The particle shapes and the AFM appearance are evidence of the formation of micelle-like structures in the solution, which probably have less soluble EDOT moiety as a core (see Table S2 for solubility parameter values) and OCL as protecting shell. These particles are expected to have enhanced stability as the OCL chains are more probable into a stable, extended conformation mediated by the hydrogen-donor character of Chl (its acidic nature), which generate favorable hydrogen-bonding interactions (cross-associations) with the nucleophilic ester groups of basic nature in OCL [62]. It appears that, from the point of view of chain conformation, the interaction of OCL with Chl has a similar result to that of the water interaction with PEG, which was explained in detail in our previous report [55]. 

The net selectivity of Chl for OCL endows the system with increased segregation power. This is because, besides solvophobic/solvophilic interactions, the π–π interaction of the EDOT rings in the core of the assembled structures are also favored due to the thiophene rings’ propensity for π-stacked dimer/oligomer formation through intermolecular interactions [83].

The **EDOT-PCL** particles in the dry state, which are discernible in the AFM images in Figure 3, are larger in size than the values of D_h_ obtained in the solution from the DLS measurement. This could be caused by the fusion of the smallest particles during solvent evaporation. There is also a significant difference between the width and height of these round structures; the height is very small (5–8 nm) compared to the width (see Figure 3C,D). Thus, from a geometric point of view, they are similar to disk-shaped particles. The asymmetry of the cross-section can be attributed to particle collapse, which is due to the deflection induced by solvent evaporation. This is concomitant with its interaction with the support during film formation. Moreover, it is important to note that no characteristic forms of OCL crystallization appeared during Chl evaporation in any of the investigated areas.There were several reasons for this. As was stated before, mica is not an appropriate support to study PCL crystallization [84] because, due to the difference in the polarity between Chl and hydrophilic mica, when a solution of PCL in Chl was spun onto it, the hydrophobic PCL was dewetted thereon, thus forming droplets in order to minimize the free energy [85]. It has been explained that when such droplets form, their high surface tension prevents the crystallization of semicrystalline polymers; thus, it is difficult to make them have a lamellar morphology [86]. In our particular case, besides there being a reason similar to that mentioned above, the EDOT moieties’ intermolecular π–π aromatic interaction, in the inner core of the micelles, could also be an effective hindrance of the crystallization of the OCL shell, as was observed by others [87]. The morphology of the thin film obtained from the **EDOT-PCL** chloroform solution on mica is in stark contrast to that obtained previously for another PCL-containing macromonomer (2,5-dibromo-1,4-phenylene-PCL substituted) in Chl [88], which formed flat-on lamellae after spin-coating on a hydrophilized SiO2 surface (without any melting and recrystallization). This behavior was attributed to the strong interaction of the PCL hydroxyl end groups with the substrate.

This comparison supports the conclusion that not only the processing conditions but most importantly, the structural peculiarities (position of the functional group—the end group—vs. “in chain” group [26]—and the type of the group) are also decisive aspects of the supramolecular organization of the functionalized oligo-/poly(ε-caprolactone) in thin films.

**Figure 4:**
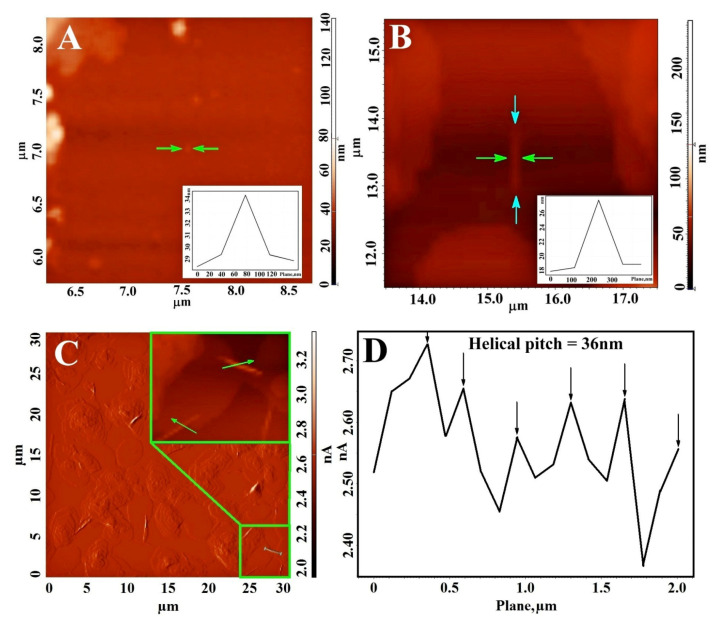
(**A**) AFM height image of round-shaped-like particles formed in the thin film of **EDOT-PCL**, obtained from its ACN solution—the inset shows a cross-sectional analysis of the particle’s horizontal line and marked by arrows); (**B**) AFM height image of a straight rod with the length marked by the blue arrows—the inset shows a cross-sectional analysis of the rod’s horizontal line and is marked by the green arrows; (**C**) AFM amplitude image evidencing the formation of the helical rods—the inset is the magnified marked area showing a high aspect ratio of helical rods with the arrows indicating both left- and right-handedness; (**D**) cross-sectional analysis that provides an average value of the helical pitch.

**Figure 5:**
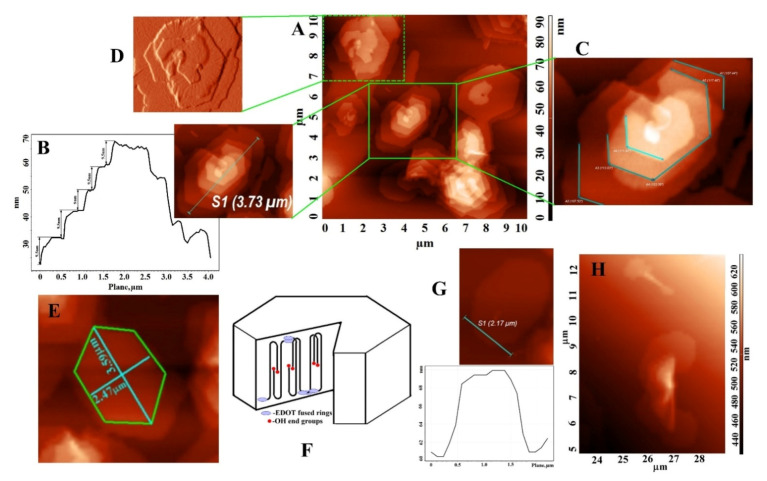
(**A**) AFM height image of **EDOT-PCL** platelets as almost perfect hexagonally shaped single crystals (SCs); (**B**) AFM height image of a multi-lamellar SC and its cross-sectional analysis, which shows individual lamellae thicknesses; (**C**) angle values between two consecutive {110} faces and between the {110} and {100} faces of SCs; (**D**) AFM amplitude image showing the usual PCL SC lamellae’s striations; (**E**) the aspect ratio of the perfect six-sided facetted **EDOT-PCL** SC; (**F**) schematic representation of possible OCL chain-folding, thereby forming the hexagonal lamella of **EDOT-PCL** SC; (**G**) AFM height image and cross-sectional analysis of a non-perfect mono-lamellar SC; (**H**) AFM height image showing straight and helical rods breakout crystallization and the starting of 2D platelet formation.

A rich and sophisticated morphology was obtained when the drop-caste film was obtained from the ACN solution (Figure 4 and Figure 5, Figures S5–S7). This is further evidence that **EDOT-PCL** behaves as a “block molecule”. This term denotes monodisperse [47,48] or slightly polydisperse [49] flexible systems that are end-capped with aromatic fragments, whose SA can be considered to be analogous to that of linear block copolymers (BCPs) constructed with one block that is significantly stiffer than the others [48]. In this context, it is important to highlight some significant aspects that are related to the self-assembly of block copolymers [89], especially the semicrystalline block copolymers that contain at least one crystallizable block. This is because crystallizable OCL is the major component of **EDOT-PCL**.

It was experimentally confirmed that, by substituting a block in a coil-coil BCP with a crystallizable one, the SA proceeds on a distinctive path, which leads to a particular behavior in the solution and in bulk [89,90,91,92], in addition to other complicated phenomena during thin film formation on a solid support [89,92]. Various interrelating parameters (such as solvent “quality”/selectivity, solution concentration, melting and crystallization temperature, the support’s surface tension, polymer–support interaction, and film thickness, to enumerate only a few) are involved in all of these circumstances [20,89,90,91,92,93]. This is because, besides the microphase separation of incompatible building blocks of BCPs, crystallization arises as a competing/complementary SA driving force, which influences its phase structure development. The competition between the two-phase transitions leads to confined, templated, or breakout crystallization modes [20,89,91,92].

In particular, self-assembling in the solution of semicrystalline BCPs is a very important topic [89,90,91,92,94] because micellar structures, which form when such BCPs are exposed to energetically unfavorable conditions for the crystalline block (a non-selective, marginal solvent), generally tend to form nanostructured morphologies with low curvatures (cylinders, ribbons, and platelets) [95]. This gives rise to nanoconfined systems, which significantly affect various stages of crystallization, thereby increasing complexity and concomitantly and dramatically changing the properties of these materials [20,92].

Moreover, the interplay between microphase separation and crystallization in the micellar systems of BCPs is also important in the context of solution-based thin film formation [96]. This is because the morphologies obtained in thin films (also 1D nanoconfined systems) are often driven by self-association in the solution [97,98].

All of the briefly mentioned aspects above touch on the behavior of **EDOT-PCL**, which in an ACN diluted solution, form micelle-like particles, with a crystallizable OCL as the core and rigid planar cycles in the shell, as was suggested by the NMR, UV-vis, fluorescence, and DLS results. In the AFM investigated area, as shown in Figure 4 and Figures S5 and S6, a co-existence of spherical-, straight rod- and helical rod-like morphology can be seen, with a small incidence of the first two in comparison to the helical rods, which appeared more frequently.

There could be many reasons for the mixed morphology of the structures, which differ in the interfacial curvature that appeared, but most likely is the OCL polydispersity index [99,100].

We attributed the formation of such structures, which have thermodynamic stability that is high enough to retain the shape after solvent evaporation, to **EDOT-PCL** microphase separation in the ACN, which was driven by the solvophobic/solvophilic interactions, which in this case dominated the structural self-organization. Our claim is supported by the large difference between the affinities of the structural components of **EDOT-PCL** in relation to ACN. Besides the recognized marginality of ACN for PCL [62], the high miscibility of EDOT with ACN can be seen because both the dipole moment and solubility parameters of EDOT (see Table S2) are simultaneously closer to those of ACN [101], as compared to OCL. The fact that a spherical micellar shape is preserved in the films (Figure 4A) is proof that solvophobic interactions overwhelm crystallization ordering in the micelles’ inner core and that crystallization could take place in the confined spaces formed by them [89,92]. This hypothesis is also supported by the fact that confinement reduces the crystallization ability of the polymer [89], which, for PCLs functionalized with bulky end groups, is significantly affected [102]. Additionally, it is well known that the core of the 3D-confined spherical micelles has the lowest degree of crystallinity, as compared to the 1D and 2D types of confinement geometries [90,91].

At this stage of our research, it is not possible to clearly state whether the core of the round micelles, formed at room temperature, is amorphous (melted) or has a degree of crystallinity. In a future study, for example, through DSC measurements in solution, the melting and crystallization temperatures of OCL could be determined in order to clearly confirm its state at room temperature.

However, it is reasonable to suppose that, due to confinement, the values of those parameters could be significantly lower than those for **EDOT-PCL** in bulk (T_m_ = 50 °C and T_c_ = 27 °C) [42]. This is similar to what was reported previously for other bulky end-groups functionalized PCL [102].

Straight rod-like structures (Figure 4B), whose presence is very likely in the solution (the DLS trace in Figure S4A is multimodal), could have formed as a consequence of the propensity of crystallizable OCLs to induce low curvature structures, in this case, the 1D rods with lower energy than spherical micelles. These rods are no more than 1 μm in length and have a width of 250 nm and a height that varies from 9 to 10 nm (Figure 4B). Even if no visual evidence has been found in AFM images, the possibility of the formation of this type of entity by the spherical particles during solvent evaporation should not be completely ruled out.

Thus, it could be possible that, during solvent evaporation, the EDOT-containing shell of the spherical micelles’ corona shrinks and thus occupies less space at the surface of the OCL core. Consequently, the spherical micelles may become unstable, and objects with a smaller overall curvature (rods) could form.

Interestingly, as compared to round particles and straight rods, there is currently a large number of visible helical-rod-shaped supramolecular assemblies with left- or right-handedness (Figure 4C), and with a width of approximately 250 nm, a length of around 2.5–3 μm, and a helical pitch of 36 nm (Figure 4D). The helical structure is a sophisticated, ubiquitous motif that is found in biological systems, the conformation of which is related to the chirality that is caused by supramolecular assembling. In some synthetic systems, helical supramolecular assemblies can be formed from highly anisotropic *achiral* amphiphiles, which contain rigid aromatic moieties [71,103,104,105,106], without external symmetry-breaking inducing factors. In all these cases, but especially for block copolymers [71,105,107], the occurrence of symmetry breaking is subtle and depends on both the molecular structure and external environmental factors.

Thus, helical assembly formation, caused by the coiling of cylindrical objects (micelles, fibrils) [105,107], was ascribed, in principle, to the introduction of an attractive interaction along their periphery, allowing for efficient contact between the surfaces of neighboring loops along the helix. There may also be other factors that could have, synergistically, contributed to the formation of the helix.

In the current system, from the three types of interactions at play during film generation, i.e., solute–solvent, solute–support, and solute–solute, the last two could be responsible for the coiling of the rod-like structures that are longer than 1 μm in a helical shape. Thus, dispersed SA structures, when deposited, wet the mica surface by exposing the EDOT rings of their shells to the surface of the support. By evaporating the ACN solvent, the solute–support interactions are increased, and in this way, the EDOT moieties from some of the **EDOT-PCL** molecules could be “stuck” to the support. There is a favorable phenomenon of complexation between K^+^ ions that naturally exist on the mica surface and the ethylenedioxy ring in EDOT, which could promote the aforementioned sticking. Such an interaction, recalling the high affinity of PEG to mica [85], could be strong enough to induce surface tension which causes helical twisting of the straight rods. The intermolecular, directional π–π stacking of aromatic EDOT rings could contribute to this bending through an anisotropic alignment of chromophores that concomitantly stabilize the formed loops. Thus, rod-like helical structures were caused by the synergy of factors that functioned as symmetry-breaking during solvent evaporation, such as the geometric shape and length of supramolecular structures, surface characteristics of the support, ion complexation, and π–π stacking interactions.

All the structures described above are metastable and “frozen” intermediate morphologies that “talk” about the events that took place during the formation of the **EDOT-PCL** film from the ACN solution. They also provide a clue to understanding the phenomena behind the formation of lamellar 2D structures. As Figure 5, Figures S6 and S7 show, most of these structures, which abundantly populate the film’s surface, have the characteristic shape of PCL single crystals (SCs).

Under various conditions, it is well established that PCL tends to form six-sided hexagonal crystals [108]. The techniques used for SCs production contain several steps, they are time-consuming, and they are generally very elaborate. For instance, freestanding PCL SCs have been obtained from solution by isothermal crystallization [109,110], by self-seeding [110,111], or by nanoprecipitation methods [112,113], and they were also formed at the interface with a solid support caused by isothermal crystallization from the melted PCL thin films [114,115]. The only PCL SCs obtained by solvent-evaporation-induced crystallization were those at the interface of two immiscible liquids [116]. To the best of our knowledge, no PCL SCs obtained in thin films on a solid support through simple evaporative crystallization from the solution at room temperature have been reported before. In Figure 5, this type of structure is as obvious as in Figure S7, which shows AFM micrographs of 30 μm × 30 μm; the areas marked in green are extended and explained in Figure 5. They are multi-lamellar, as in Figure 5A, or mono-lamellar, as shown in Figure 5G (the expanded area from Figure S7B). They are almost geometrically regular and are placed in a *flat-on* fashion in relation to the support surface. Generally, this type of lamellar orientation is the preferred one for thinner films of semicrystalline polymers [92], while for linear PCLs, it was observed in crystal growth in molten-state ultrathin films [114]. It is common that the film thickness can be adjusted by the concentration of the solution used for casting [89]. As we used to process our films, a concentration of the solution with a value comparable to that of the linear PCLs, which formed films with thicknesses ranging from 1 nm to 200 nm [114], it can be assumed that the thicknesses of the **EDOT-PCL** films, whose morphology is shown in Figure 5, Figures S6 and S7, does not exceed the upper limit of the mentioned range.

However, an uneven thickness of films can also be seen in these figures. As can be observed in Figure 5A, the SCs with micrometric lengths have the usual PCL lamellar morphology, with large base platelets that are covered with several lamellae as hexagonal terraces, which grow from a screw dislocation [109,110,112,115,117]. The thickness of individual lamellae is around 9–10 nm, as was obtained from the measurement of the cross-section trace shown in Figure 5B, which is typical for linear PCL SCs [117]. A similar thickness value was also detected for monolamellar SC presented in Figure 5G. Defined as one of the most important types of defect in polymer structures [118], the screw dislocations found in both PCL-containing linear and branched architectures [110,112,117,119] forbid the crystallographic coherence between successive layers, which give rise to spectacular rotations that are always associated with a pyramidal habit, as can be seen in the bottom-right corner of Figure 5A. These screw dislocations have been attributed to different factors [120], but as structural factors, the non-planarity of the ester group carbonyl in the PCL repeating units and the effect of chain ends [118] (especially bulky ones) have been cited as the main reasons.

PCL SCs are constituted by four {110} and two {100} growth faces, with theoretical values of angles between two consecutive {110} faces and between the {110} and {100} faces of 113° and 124°, respectively [117,119]. Through AFM measurements, the values of both types of angle were determined for the three layers of the hexagonal lamellae of **EDOT-PCL** (Figure 5C). For the angle between the {110} and {100} faces, the value of 123.59° was very close to the theoretical one; for the angles between two consecutive {110} faces, the values only slightly deviated from 113° (113.03° and 111.9°). It is worth noting that similar angles values were reported for linear PCL with a degree of polymerization of 17 [117] and for a star-branched structure with PCL arms having a degree of polymerization of 10 [119]. These structures had a degree of polymerization for PCL close to that of **EDOT-PCL**.

The growth faces sectoring of the SC lamellas is partially visible in the AFM amplitude micrograph in Figure 5D; it is clear enough to attribute this, with certainty, to the existence of the six crystallographic faces of **EDOT-PCL** SC. Moreover, in the same figure, the striations that are typical of PCL SCs [121], running from the center to outwards, give an evident contrast and reveal the different sectors. Surface striations are common features for both linear and branched structure-derived PCL SCs [117,119], which have received various explanations. They have been attributed to the presence, in the PCL SCs, of micro-crystals with a nano-order size that was oriented along the growth direction [110], or they originated from the non-planar crystal structure [122].

**Figure 6:**
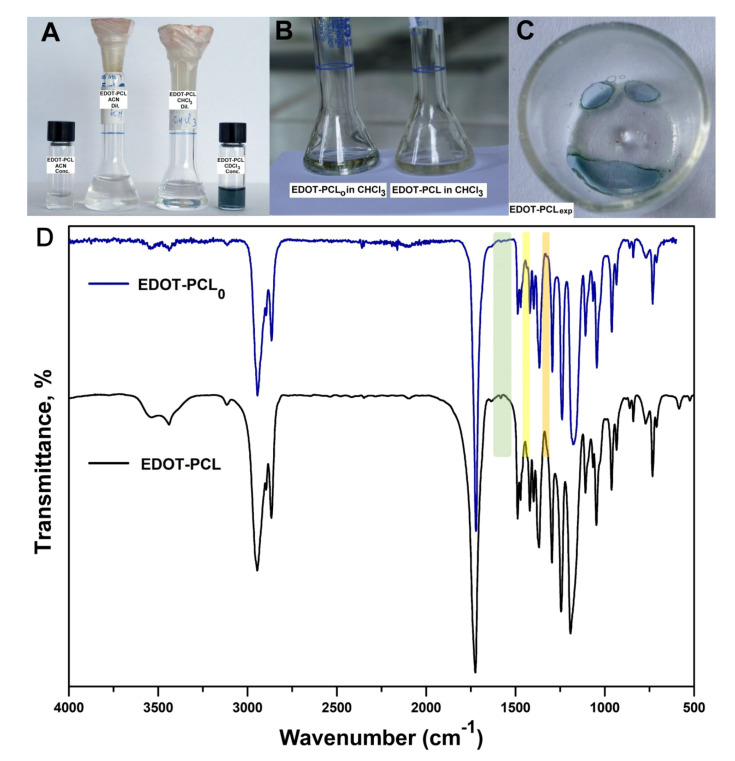
(**A**) (From left to right): the **EDOT-PCL** in an ACN concentrated solution, in an ACN diluted solution, diluted **EDOT-PCL** in Chl, and very concentrated CDCl_3_ solution of **EDOT-PCL**; (**B**) **EDOT-PCL_o_** and **EDOT-PCL** in the Chl-concentrated solution; (**C**) blue film layer appeared after evaporation of the chloroformic solution of **EDOT-PCL** treated with hydrochloric acid (HCl) (**EDOT-PCL_exp_**); (**D**) FT-IR spectra of **EDOT-PCL** and **EDOT-PCLo**.

The aspect ratio of the SCs, defined as the ratio of the value of the long axis (l_b_) and the short axis (l_a_), is an important SC parameter, and provides an indication of the crystal growth rate along these axes. The l_b_ and l_a_ values are given in Figure 6E (the expanded area from Figure S7A) for an almost perfect six-sided and facetted **EDOT-PCL** SC, which resulted in a shape factor of 1.45; the latter characterizes a PCL SC that is less anisotropic than the one resulting from the linear PCL with a degree of polymerization of 17. This was obtained by isothermal crystallization from the n-hexanol and had a shape factor of 2 [117].

On the issue of the formation of semicrystalline polymers SCs, they resulted from the bottom-up self-assembling process of crystallization [89], involving polymer chains folding back and forth within the lamellae [116]. By using the information for the individual lamella thickness given by the AFM height scanning profile in Figure 5B,G (an average value of 9.5 nm), combined with the degree of polymerization of 16.5 for extended OCL length [42], and considering the PCL unit cell parameters [69,117], the number for non-integral chain folding was 0.5. We attempted to develop an idea about the arrangement of the **EDOT-PCL** chains inside the lamellar SCs using this value, and these are represented in the sketch in Figure 5F. This drawing, therefore, shows hexagonal lamella that was formed by non-integral, once-folded OCL chains with adjacent re-entry. We considered this arrangement to be more probable, in comparison to a tilted extended-chain crystal [123], motivated by the high affinity of the EDOT ring to the mica support and by the preference of OCL (in itself an amphiphile [69]) to migrate towards the film‘s surface from the ACN marginal solvent. Thus, it exposed its aliphatic part towards the film’s surface but kept the OH polar hydrophilic chain ends inside the solution for a longer period of time during solvent evaporation.

Hypothetically, interchain hydrogen bonding has also been suggested to be ideal in the solid state.

It is obvious that all of the above described and characterized crystalline 2D lamellar structures derived from the SA structures formed in the solution. Their formation in the film was mediated by ACN evaporation and the **EDOT-PCL** interaction with the hydrophilic mica support. These two basic phenomena conducted modification in the corona of micelle-like SA structures that were formed in the solution. Such modification most probably triggered the alteration of the micellar structures, leading to further morphological transitions toward organized structures of the 2D lamellar SCs into the new confined space of the film. In fact, the segregation strength of the system in the solution is affected by ACN evaporation, and OCL breakout-like crystallization is possible, which disrupts the micelle core, leading to lamellar SCs [91]. In support of this statement, the AFM image presented in Figure 5H (the expanded area from Figure S7C) shows the obvious breakout-like transformation of the straight and helical rods in addition to the development of crystal growth front of the 2D platelet’s formation. At this point, it could be speculated that the multi-lamellar structures could be formed by rod transformation, while an almost oval, less-defined mono-lamellar structure (as is presented in Figure 5G) derived from the spheroidal micelles. Moreover, in Figure S7D, the green arrows mark the presence of rods, especially helical rods, in the immediate neighborhood of the hexagonal multilayered **EDOT-PCL** SCs; the image shows a “frozen” stage of such rods, which coalesce during solvent evaporation and film formation and their transformation into a more thermodynamically stable SCs. It could be assumed that, in the case of **EDOT-PCL**, as is the case for other microphase-separated asymmetric diblock copolymers [124], a structural relaxation of OCL takes place during solvent evaporation, inducing disintegration of the microphase separated structures, thus allowing for breakout crystallization and the formation of SCs. In view of all these phenomena, the role of the ACN solvent should not be overlooked because it was reported that a poor solvent could function as a nucleating agent able to induce PCL ultrathin film crystallization [84].

The AFM investigation allowed us to demonstrate the SA of **EDOT-PCL** in the two different solvents, as well as to highlight, in the case of ACN, an “order-to-order” type transition in confined spaces (from the dispersion to the thin film), which is mediated by microphase separation and crystallization, thus demonstrating the ability of **EDOT-PCL** to behave as a “block molecule”.

### 3.3. EDOT-PCL Behavior in the Presence of Acids

The phenomena and the experiments described in this section were generated by an unexpected event that occurred during the preparation of the **EDOT-PCL** concentrated solution in CDCl_3_ for ^13^C-NMR registration. More precisely, a blue color appeared, the intensity of which increased with the increase of the **EDOT-PCL** amount added.

First, we supposed that the appearance of the specific blue color of PEDOT was a consequence of the self-assembling of **EDOT-PCL** in CDCl_3_, which was due to the increase in the solution concentration, thereby forming a supramolecular, linear-like conjugated chain. The phenomenon of a color appearance, when the solution concentration was increased, was also observed by others and was explained in this way [125]. It is important to note that, in the ^1^H-NMR spectrum of this blue solution (performed at an **EDOT-PCL** final concentration of 55 mg/mL), there were no obvious changes, as compared to the one recorded at a lower concentration (6.7 mg/mL) (shown in Figure S1B). This highlights that the appearance of the blue color should be due to other causes. Thus, we went further with the assumption that, in ACN (which is a marginal solvent for PCL), this alleged self-assembling phenomenon could be hindered/affected as PCL has a collapsed conformation, and consequently, regardless of whether it is diluted or concentrated, the ACN solutions should be colorless. As can be seen in Figure 6A, which shows the small bottle and the Erlenmeyer flask on the left side of the image, this supposition was experimentally sustained when solutions, with diluted or concentrated **EDOT-PCL** in ACN, were prepared.

In order to add supplementary evidence referring to the influence of OCL conformational state (extended or collapsed) on the supramolecular SA, we dissolved a small amount of the dark blue solid, denoted as **EDOT-PCL_o_** (which was obtained after the natural evaporation of CDCl_3_—Figure S8A), in dry acetonitrile. In contrast to the CDCl_3_ solution, this **EDOT-PCL** solution is colorless and transparent (see Figure S8B). The only explanation for this difference in solution color could be due to the influence of the collapsed conformation of the OCL side chains on the short conjugated chain of PEDOT that supposedly was formed (vide infra).

In the next step towards understanding the appearance of the **EDOT-PCL** blue color in CDCl_3_, we tried to reproduce the facts that were experimentally found during the dissolution of **EDOT-PCL** to make sure that this phenomenon is reproducible and, eventually, to determine the **EDOT-PCL** concentration at which the blue color appears.

Thus, using pure grade chloroform from a recently opened bottle, we prepared a new concentrated solution of **EDOT-PCL**. Surprisingly, the solution remained colorless no matter how much of the macromonomer we dissolved (see the flask on the right side of Figure 6B). Thus, the conclusion was that the appearance of the blue color in the CDCl_3_ in the NMR registration tube, the intensity of which increased with the added amount of **EDOT-PCL**, was not related to the formation of a supramolecular polymer by SA but could be due to another reason. Thus, we drew our attention to the well-known fact that deuterated chloroform presents an acidic character during storage for longer than 6 months [126]. The presence of acidic protons (H^+^) is relevant because, as it was noted previously, EDOT and a limited number of its derivatives have the mechanistically important capacity to form an active electrophilic intermediate [EDOT-H]^+^. This intermediate is able to initiate oligomerization [127]. If the presence of the acidic protons (H^+^) in CDCl_3_ is accepted, this means that what really happens in the NMR tube could actually be a spontaneous oligomerization reaction of **EDOT-PCL** in the absence of an oxidant but in acidic media. This is a way for EDOT or EDOT derivatives polymerization that is not so often reported about in the literature [128,129]. Starting from this supposition, we added several drops of concentrated hydrochloric acid (HCl) to the colorless, transparent **EDOT-PCL** solution in CHCl_3_. The color of the solution remained unchanged for several hours, but we found that a blue pellicle appeared in the vessel the next day, after an overnight reaction, and the solvent evaporated (Figure 6C). This new experimental finding allowed us to conclude that an oligomerization reaction of **EDOT-PCL** could have taken place in the NMR tube during the attempt to increase the concentration of the solution for ^13^C-NMR registration. In order to check for the possibility of oxidant-free, H^+^-mediated **EDOT-PCL** oligomerization, FT-IR spectroscopy analysis of the blue solid **EDOT-PCL_o_** and UV-vis and fluorescence spectroscopies of **EDOT-PCL_o_** in Chl solution were chosen. The obtained results are presented in Figure 6D, Figure S3A, and Figure 2.

From the IR spectra presented in Figure 6D, the differences between the compared compounds are obvious. The most important difference was registered in the region of 1650–1300 cm^−1^, which is specific for the characteristic absorptions that originate from the asymmetric and symmetric C=C and symmetric C-C stretching vibrations of the aromatic thiophene ring [129,130,131,132]. Significant modifications also appeared for the peaks that are specific to poly-ε-caprolactone. Generally, a shifting of the peak positions toward wavenumbers with a lower value can be observed. This type of shifting is similar to that which was observed for other thiophene compounds during the transition from monomer to polymer [132]. Thus, in our spectra in Figure 6D, the peaks from 1637 cm^−1^ and 1584 cm^−1^ in **EDOT-PCL** changed the position to 1635 and 1581 cm^−1^ in **EDOT-PCL_o_**. Moreover, a new and shallow but discernible absorption peak, centered at approximately 1553 cm^−1^, appeared in the spectrum of **EDOT-PCL_o_**, which could be attributed to newly formed conjugated sequences. Additionally, the presence in the **EDOT-PCL_o_** blue compound of two new small but sharp, individual peaks at 1437 cm^−1^ and 1327 cm^−1^, characteristic of PEDOT [130,133], was observed. The most significant shifting from 1192 cm^−1^ to 1176 cm^−1^ was that of the band attributed to both PCL and the ethylenedioxy group in EDOT for -CH2 deformation and C–O–C bending vibration [42]. This shifting could be evidence for a vicinity change of ethylenedioxy-ring in EDOT moiety, caused by the possible appearance of a new short conjugated PEDOT sequence. In Figure S3A and in Figure 2B, the photophysical properties of **EDOT-PCL_o_** are presented. The measurements were performed in Chl at the same concentration as for **EDOT-PCL**. It can be seen that, in comparison to those of the **EDOT-PCL**, a different absorption for **EDOT-PCL_o_** appeared in UV-vis at 345 nm, together with other shallower ones at 380, 425, and 445 nm (identified in the original spectrum and given in Table S3). For the fluorescence, a noticeable increase in intensity alongside a significant bathochromic shift of the emission maxima (placed in the 400–522 nm range; see Table S3 for exact values) appeared. These findings more obviously demonstrate that **EDOT-PCL** evolved into an oligomerized form during dissolution in CDCl_3_, and this was promoted by the presence of the acidic protons.

This assumption is also supported by the recent claim by Tomsik et al. that protons could have a catalytic effect on the oxidant-free self-polymerization of EDOT and other thiophene derivatives, as they showed that, in the case of EDOT, when the reaction took place in deuterated acetic acid, no self-polymerization occurred [129]. This type of synthesis would be of great interest in terms of finding more sustainable polythiophenes, especially those designed for bioapplications, and this deserves future study.

## 4. Conclusions

In summary, we have presented a ε-caprolactone oligomer that is end-functionalized with an aromatic π-conjugated EDOT moiety (**EDOT-PCL**). Synthesized by the ROP of ε-caprolactone, **EDOT-PCL** was designed as a functional and reactive “hydrophobic amphiphile” and was shown that it could have an interaction-based bias in selective organic solvents. Thus, the non-covalent interactions, which favor self-assembling, are encoded in its structure by the presence of π-conjugated thiophene ring in the EDOT moiety as well as due to hydroxyl end groups of OCL chains.

The experimental results demonstrate that the self-assembling of **EDOT-PCL** in the solution and the morphology in thin films are balanced by the mismatch between aromatic-aliphatic nature, rigid-flexible character, and between the packing geometry of its structural building elements. Besides these structural peculiarities, the **EDOT-PCL** SA is determined by the solute–solute, solute–solvent, solute and/or solvent–support interactions assisted by weak non-covalent forces (such as solvophobic–solvophilic character, π–π interactions, ionic complexation, and hydrogen bonding). Additionally, due to the semicrystalline character of OCL, the bottom-up hierarchical self-assembling of **EDOT-PCL** is governed by both driving forces, i.e., microphase separation and crystallization. Thus, in both solvents and in the thin film obtained from the Chl solution, SA in the nanoscale morphologies was predominantly driven by phase separation, which overwhelms crystallization ordering. It is important to note the metastable nature of the micelle-like nanostructures that are formed in the ACN solution when they were drop-cast in the thin film on mica. Due to solvent evaporation, which triggered the bulky EDOT moiety rearrangement through its particular interaction with the support, the OCL crystals were no longer confined to the nano-/microdomains in which they nucleate, and breakout crystallization occurs. This unexpectedly led to the development of 2D lamellar SCs at room temperature without any supplementary treatment. The AFM investigation revealed, in the case of ACN, an “order-to-order” transition in confined spaces (from dispersion to thin film), mediated by microphase separation and crystallization, thus demonstrating the ability of **EDOT-PCL** to behave as a “block molecule” in a similar manner as shape amphiphiles, with an essential contribution of the EDOT end-group.

Moreover, the self-assembled supramolecular structures are fluorescent, with increased intensity in less polar chloroform solvent in comparison to ACN. The OCL shell preserved, in this case, the fluorescence of the EDOT moiety. This event is illustrative of the EDOT moiety’s impact on the OCL properties, which is dependent on the EDOT placement inside or outside of a confined space into a supramolecular structure. It was shown that EDOT confinement also impacted the crystallization behavior of OCL in a thin film on a support of hydrophilic nature.

Exposing **EDOT-PCL** in Chl to inorganic HCl aqueous solution proposed an answer for the blue color, which unexpectedly appeared during macromonomer dissolution in CDCl_3_ of acidic nature. **EDOT-PCL**’s oligomerization due to solvent’s acidic character could be a preliminary presumptive explanation. In the future, a deep study dedicated to oxidant-free polymerization of **EDOT-PCL** in the presence of various acids is intended in order to systematically investigate the optimal conditions for synthesis and to fully characterize the eventually obtained PEDOT graft polymer. This method could become a more sustainable pathway for biocompatible PEDOT synthesis.

## Data Availability

Tha data presented in this study are available on request from the corresponding authors.

## Supplementary Materials

The following are available online at https://www.mdpi.com/article/10.3390/polym13162720/s1. Explanatory details; discussion on the ^1^H-NMR and ^13^C-NMR of **EDOT-PCL**; calculation of OCL’s theoretical diameter of gyration D_G_; Table S1: Chemical shifts (ppm) characteristic to ^1^H-NMR spectra of **EDOT-PCL** recorded in CDCl_3_ and CD_3_CN; Table S2: Some physical properties of the used solvents and those related to constitutive parts of **EDOT-PCL** macromonomer; Table S3: Photophysical characteristics of the investigated compounds in solutions; **Figure S1**. ^1^H-NMR spectra of **EDOT-PCL** macromonomer in (**A**) CD_3_CN and (**B**) CDCl_3_; **Figure S2**. **EDOT-PCL** 2D NOESY ^1^H-^1^H experiment registered in CD_3_CN at concentration of 6.66mg/mL (whole registered range); **Figure S3.** (**A**) UV-vis spectra of **EDOT-PCL** macromonomer and of its oligomerized form (**EDOT-PCL_o_**) in indicated solvents at concentration of 1 mg/mL, (0.49 × 10^−6^M); (**B**) UV-vis spectra of **EDOT-PCL** macromonomer registered at concentration of 0.5 mg/mL (0.245 × 10^−6^M); (**C**) UV-vis traces of **EDOTMeOH** in Chl and ACN; (**D**) fluorescence spectra of **EDOTMeOH** in Chl (λ_excit_ = 262 nm) and ACN (λ_excit_ = 254 nm); **Figure S4**. DLS traces of **EDOT-PCL** in Chl and in ACN solutions, at the concentration of 1mg/mL as (**A**) intensity-weighted distribution of apparent hydrodynamic diameter (D_h_) and as (**B**) volume-weighted distribution of D_h_; **Figure S5**. AFM height images of **EDOT-PCL** films, obtained by drop-casting of its ACN solution on mica, showing round-shaped particles: (**A**–**C**) successively magnified areas, marked with the green color, in each image; **Figure S6**. AFM height images of **EDOT-PCL** films, obtained by drop-casting of its ACN solution on mica, showing round-shaped particles: (**A**–**C**) successively magnified areas, marked with the green color, in each image; **Figure S7**. AFM height images of **EDOT-PCL** films, obtained by drop-casting of its ACN dispersion on mica, used for morphology characterization as detailed in expanded format in Figure 5 (green colour marked areas): (**A**) a perfect six-sided facetted **EDOT-PCL** single crystal (SC), expanded in Figure 5E; (**B**) a non-perfect mono-lamellar **EDOT-PCL** SC, expanded in Figure 5G; (**C**) straight and helical rods’ breakout crystallization and starting of 2D platelets formation; (**D**) green arrows showing the rods (most of them in helical shape) placed in the immediate neighborhood of the hexagonally shaped, multilayered **EDOT-PCL** SCs; **Figure S8**. (**A**) Photo showing the bulk form of **EDOT-PCL** as resulted from the reaction as an white-grey powder (middle), of **EDOT-PCL** as resulted after evaporation of an ACN solution (right) and **EDOT-PCL** oligomerized form (**EDOT-PCL_o_**), as resulted after evaporation of acidic CDCl_3_ solution (left); (**B**) photo showing **EDOT-PCL_o_** having dark blue colour in solid state (left) and the colourless, transparent aspect of **EDOT-PCL_o_** ACN solution (which shows a blue colour when is solved in chloroform—see Figure 6B in main manuscript).
