# Supplementary material for: 3,4-Ethylenedioxythiophene (EDOT) End-Group Functionalized Poly-ε-caprolactone (PCL): Self-Assembly in Organic Solvents and Its Coincidentally Observed Peculiar Behavior in Thin Film and Protonated Media
Source: Polymers (Basel). 2021 Aug 14;13(16):2720. doi: 10.3390/polym13162720 (PMC8400159; doi:10.3390/polym13162720)
Supplement: Supplementary file 1 [file polymers-13-02720-s001.zip › polymers-1291408-supplementary.pdf]

Supplementary Materials

3,4-Ethylenedioxythiophene (EDOT) end - group functionalized poly-ε-caprolactone (PCL): self-assembling in organic solvents and its coincidentally observed peculiar behaviour in thin film and protonated media

Anca - Dana BENDREA,[a] Luminita CIANGA,[a*] Gabriela - Liliana AILIESEI,[b] Elena - Laura URSU,[a] Demet Göen-Colak[c] and Ioan CIANGA[a*]

[a] "Petru Poni" Institute of Macromolecular Chemistry, Centre of Advanced Research in Bionanoconjugates and Biopolymers, 41A, Grigore –Ghica Voda Alley, 700487, Iasi, Romania; anca.bendrea@icmpp.ro; lcianga@icmpp.ro; ursu.laura@icmpp.ro; ioanc@icmpp.ro
[b] "Petru Poni" Institute of Macromolecular Chemistry, NMR Spectroscopy Department, 41A, Grigore –Ghica Voda Alley, 700487, Iasi, Romania; gdarvaru@icmpp.ro
[c] Istanbul Technical University, Faculty of Science and Letters, Department of Chemistry, Maslak, 34469 Istanbul, Turkey; goende@itu.edu.tr
*- Corresponding authors: ioanc@icmpp.ro; lcianga@icmpp.ro; Tel.: +40 232 217 454

Explanatory details

Due to the fact that different investigations/measurements/ analyses were performed at different moments and/or persons, the compounds' name written on labels attached to flasks/ bottles sometimes differed from each-other (as can be seen in some photos presented along both the paper and Supporting Materials). In order to clarify the facts, in the present report were established the general code names as follows:

- **EDOT-PCL**- the synthesized macromonomer as resulted from the ROP reaction;
- **EDOT-PCLo**- the oligomerized form of the macromonomer, having blue colour, as resulted after **EDOT-PCL** dissolution in deuterated chloroform ($CDCl_3$) with acidic character, followed by solvent evaporation;
- **EDOT-PCL$_{exp}$**- **EDOT-PCL** oligomerized by acidification with hydrochloric acid of its chloroform solution;

Discussion on the ^{1}H-NMR and ^{13}C-NMR of EDOT-PCL

In Figure 1 of the main manuscript signals at 6.37 ppm in ^{1}H-NMR spectrum and those at 100.62 ppm and 142 ppm in ^{13}C-NMR spectrum, respectively, are attributable to thiophene ring. In the region between 1.30-2.35 ppm of ^{1}H-NMR spectrum are discernible the signals corresponding to methylene protons from OCL structural units, denoted with **f**, **g**, **h** and **i** in Figure 1a, while the protons **j** in aliphatic CH_2 group, directly bonded to oxygen atom, appeared downfield shifted at 3.99-4.03 ppm. It is worthy to mention that in both proton and carbon NMR spectra of **EDOT-PCL** macromonomer, the signal corresponding to protons of CH_2 group nearest to hydroxyl chain end (protons **k** at 3.44-3.47 ppm in Figure 1a) along with signals for carbon atoms of the OCL final structural unit (**9**, **10**, **11** and **14** noticed in the region between 25-35 ppm and **15** at 62.4-62.7 ppm in Figure 1b) appeared separately from the signals attributed to the rest of oligomeric OCL structural units. Signals of quaternary carbon atoms belonging to carbonylic group of ester function appear at 174 ppm.

Calculation of OCL's theoretical diameter of gyration D_G

The value of this parameter was calculated using a formula reported in [1], as follow:

$\mathbf{D_G}$ = 2x $\sqrt{}nb^2/6$; n - is the number of the repetitive structural units, namely 16 in our case; b- is the apparent Kuhn segment length, the value of which is 0.7 nm for PCL [2]. Thus, resulted D_G = 2.28 nm.

Table S1. Chemical shifts (ppm) characteristic to ^{1}H NMR spectra of EDOT-PCL recorded in $CDCl_3$ and CD_3CN

Protons notation for ^{1}H NMR	δ (ppm) in $CDCl_3$	δ (ppm) in CD_3CN	$\Delta\delta=\delta_{CDCl3} - \delta_{CD3CN}$
a,b	6.31-6.34	6.36-6.38	-0.04
c	4.36-4.41	4.33-4.38	0.03
d	4.32-4.33	4.25-4.26	0.07
e	4.22-4.26	4.20-4.24	0.02
f	2.30-2.42	2.24-2.34	0.08
i,g	1.59-1.70	1.54-1.61	0.09
h	1.36-1.44	1.30-1.37	0.07
j	4.05-4.10	3.98-4.03	0.07
k	3.66-3.69	3.44-3.47	0.22

Table S2. Some physical properties of the used solvents and those related to constitutive parts of EDOT-PCL macromonomer

Compound	Solubility parameter(MPa)$^{1/2}$	Dipole moment(D) (b_p °C/ε)*	Reference
chloroform	19	1.04;(61/4.81)	3
acetonitrile	24.8	3.92; (82/37.5)	4
PCL	20,57	1.72†	5, 6†
PEDOT	21.3	4.37/1.87**	7,8**

*- the data in parentheses are b_p- boiling points and ε- dielectric constants of organic solvents;

† - the PCL repeat unit [–(CH2)$_5$COO–] has a dipole moment originating from the ester group (total ester dipole moment 1.72 D as obtained from dilute solutions in dioxane) with components parallel and perpendicular to the backbone of 0.64 D and 1.6 D respectively;

**- dipole moment of EDOT

Table S3. Photophysical characteristics of the investigated compounds in solutions[a]

Compound	Solvent	λ_{max}abs (nm)	λ_{max}em (nm)
	$CHCl_3$	**262**	**350**
**EDOT-MeOH**	ACN	**239**;**254**	**325**
	$CHCl_3$	**257**	**370**; **430**
**EDOT-PCL**	ACN	**239**;**254**	**365**;*395*
**EDOT PCL$_o$**	$CHCl_3$	**345**; *380*; *425*; *445*	**398**; **446**; *526*; *552*

[a] - The values written in bold are the main peaks while those in italicised format are the noticed shoulders ; the concentration of solutions was kept constant as 1 mg/mL

Supplementary figures (Fig. S1-Fig. S 10)

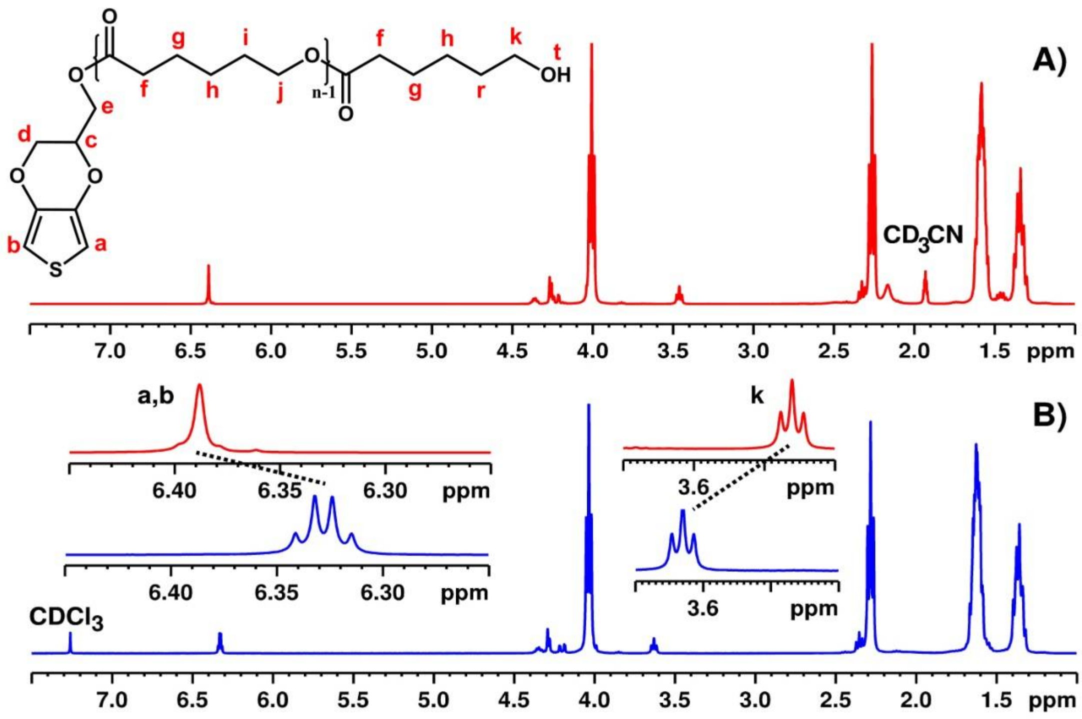

Figure S1. ^{1}H NMR spectra of **EDOT-PCL** macromonomer in (**A**) CD_3CN and (**B**) $CDCl_3$

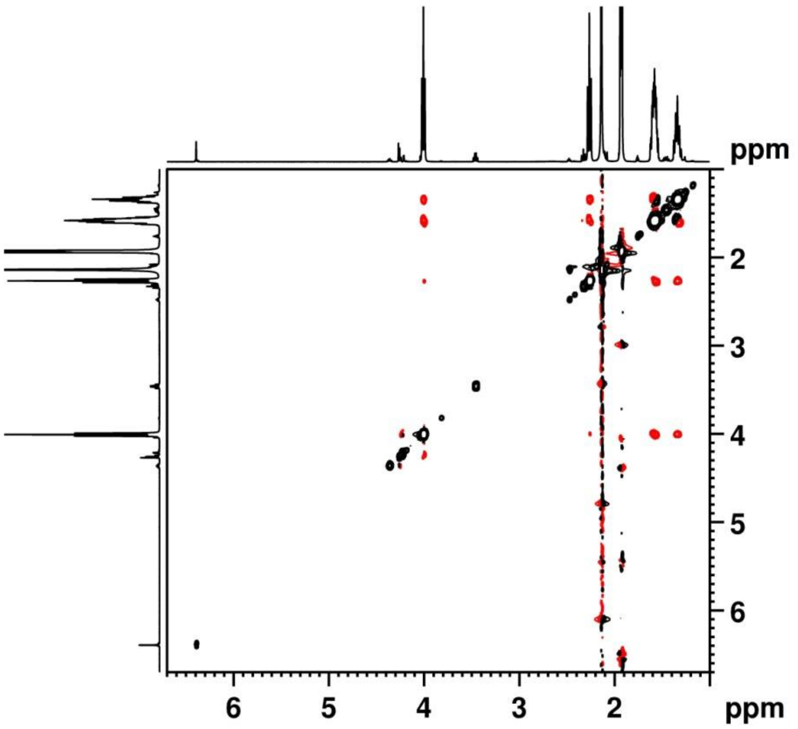

Figure S2. **EDOT-PCL** 2D NOESY ^{1}H-^{1}H experiment registered in CD_3CN at concentration of 6.66mg/mL (whole registered range)

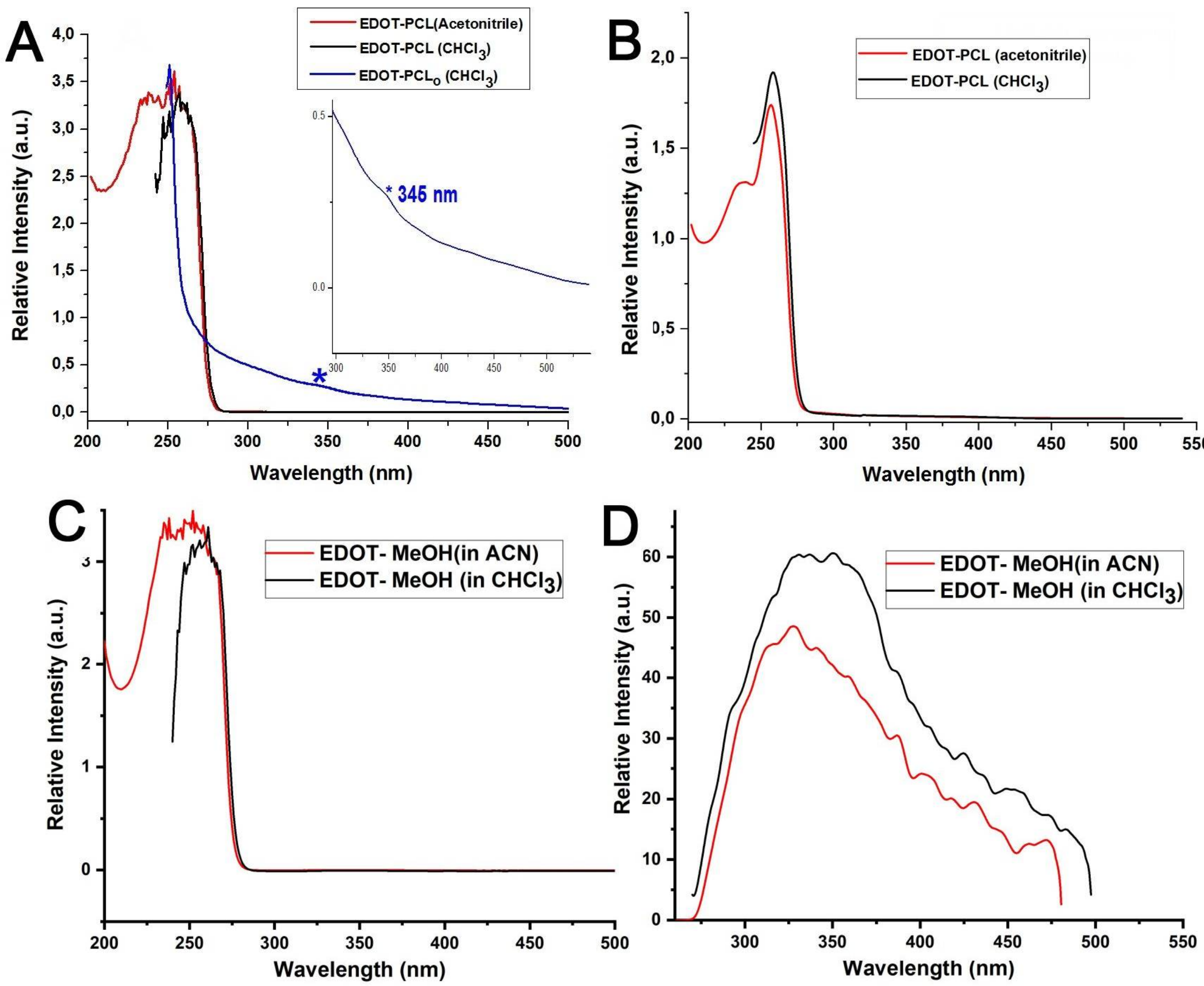

Figure S3. (**A**)- UV-vis spectra of **EDOT-PCL** macromonomer and of its oligomerized form (**EDOT-PCL$_o$**) in indicated solvents at concentration of 1 mg/mL, (0.49x10^{-6}M); (**B**)- UV-vis spectra of **EDOT-PCL** macromonomer registered at concentration of 0.5 mg/mL (0.245x10^{-6}M); (**C**)- UV-vis traces of **EDOTMeOH** in Chl and ACN; (**D**) Fluorescence spectra of **EDOTMeOH** in Chl (λ_{excit}=262 nm) and ACN (λ_{excit}=254 nm)

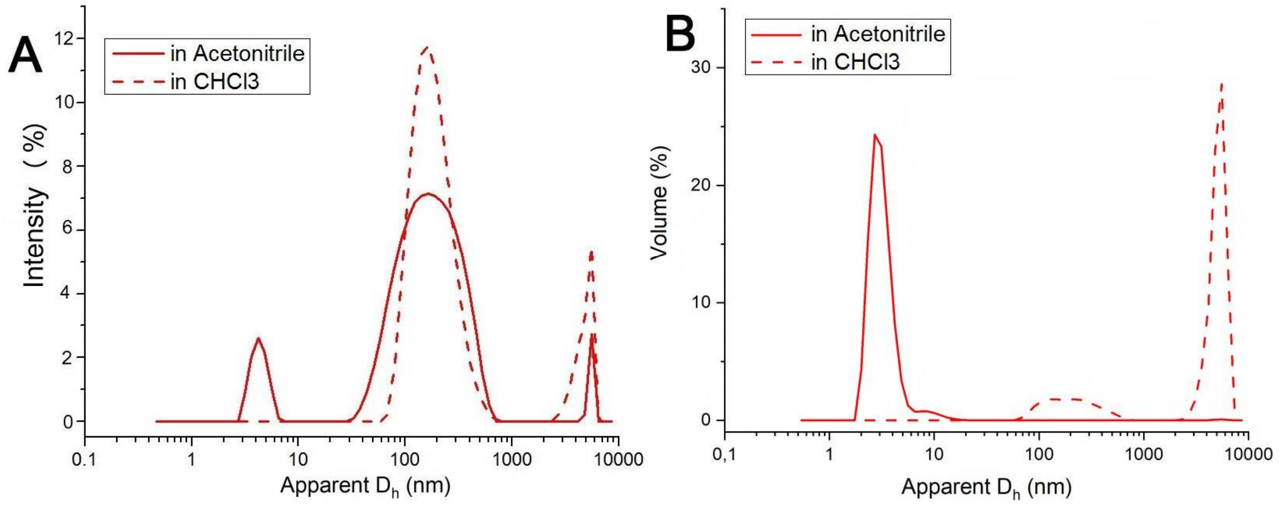

Figure S4. DLS traces of **EDOT-PCL** in Chl and in ACN solutions, at the concentration of 1mg/mL as (**A**) intensity-weighted distribution of apparent hydrodynamic diameter (D_h) and as (**B**) volume-weighted distribution of D_h

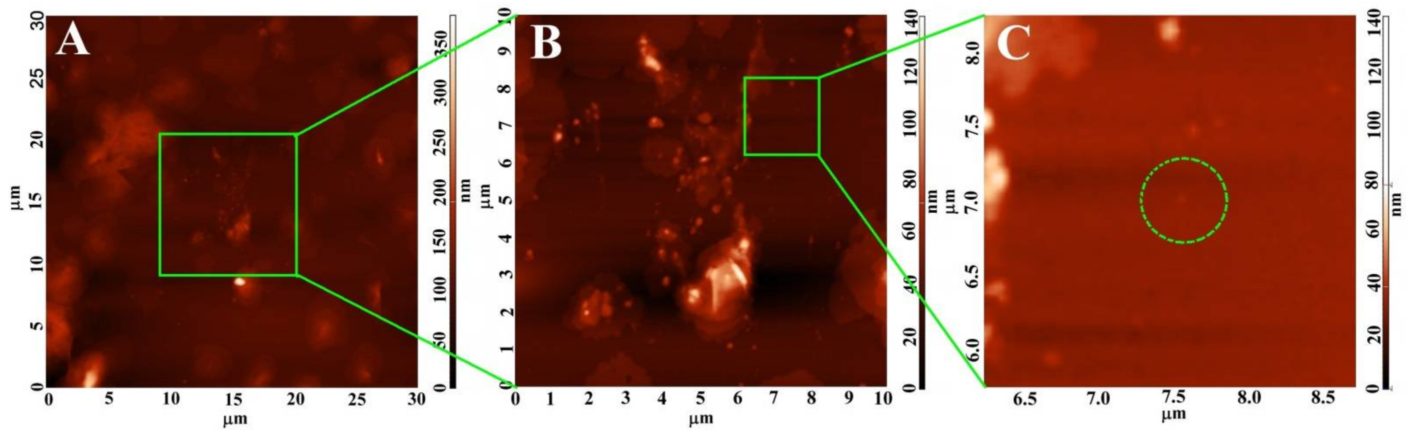

Figure S5. AFM height images of **EDOT-PCL** films, obtained by drop-casting of its ACN solution on mica, showing round-shaped particles: (**A**) (**B**) and (**C**) - Successively magnified areas, marked with the green color, in each image.

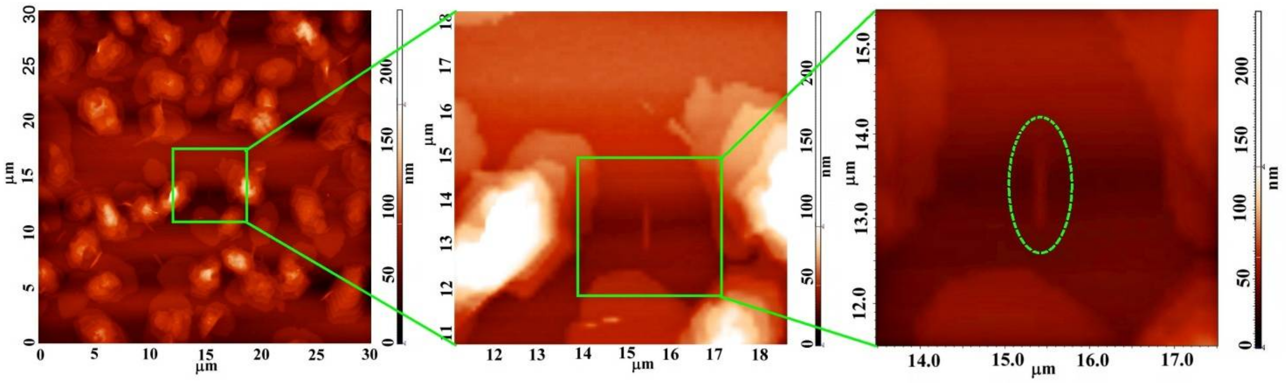

Figure S6. AFM height images of **EDOT-PCL** films, obtained by drop-casting of its ACN solution on mica, showing microphase separated straight -like rods: (**A**) (**B**) and (**C**) - Successively magnified areas, marked with the green colour, in each image.

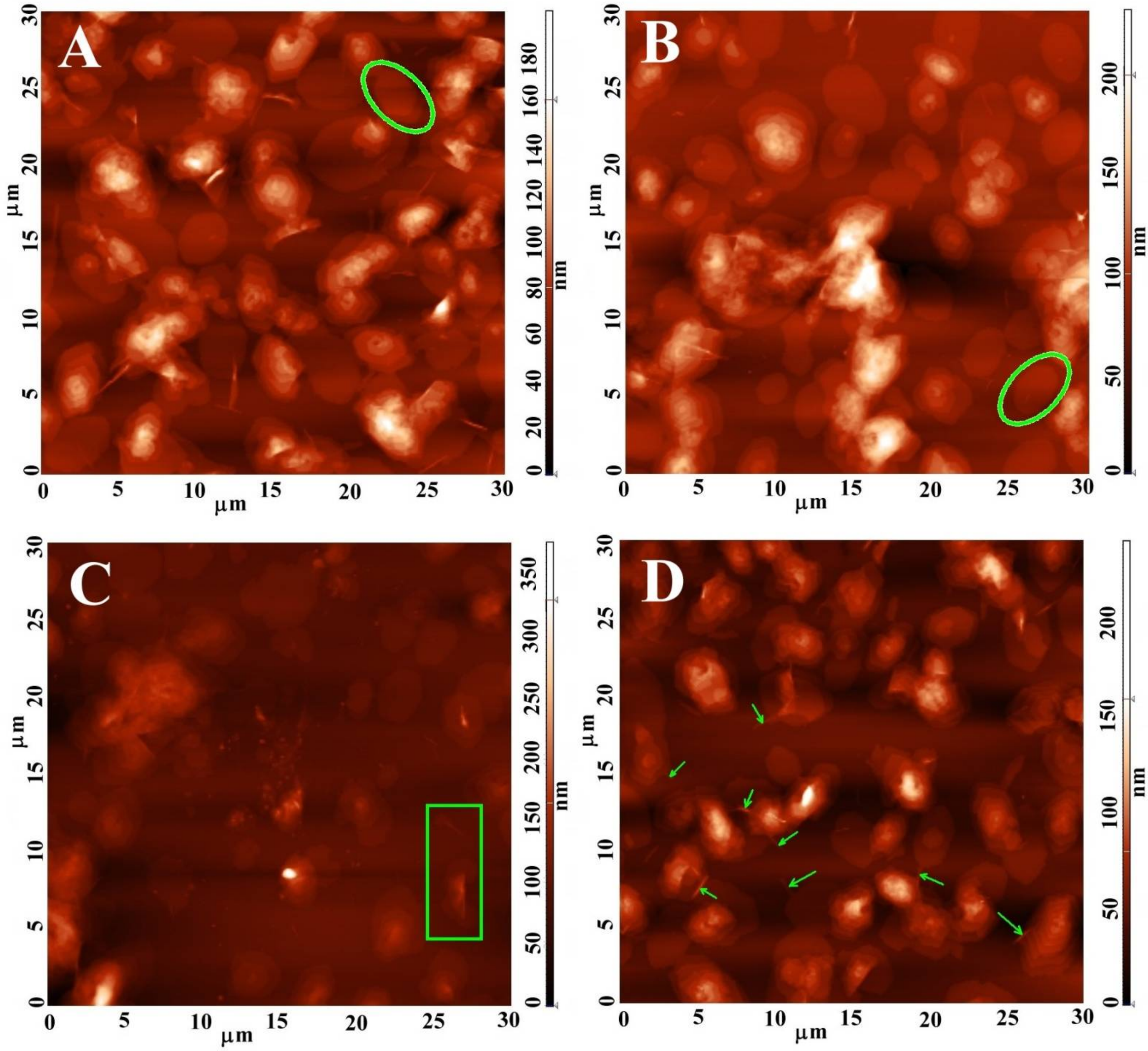

Figure S7. AFM height images of **EDOT-PCL** films, obtained by drop-casting of its ACN dispersion on mica, used for morphology characterization as detailed in expanded format in Figure 5 (green colour marked areas): (**A**)- A perfect six-sided facetted **EDOT-PCL** single crystal (SC), expanded in Figure 5 E; (**B**)- A non-perfect mono-lamellar **EDOT-PCL** SC, expanded in Figure 5 G; (**C**)- Straight and helical rods' breakout crystallization and starting of 2D platelets formation; (**D**)- Green arrows showing the rods (most of them in helical shape) placed in the immediate neighborhood of the hexagonally-shaped, multilayered **EDOT-PCL** SCs.

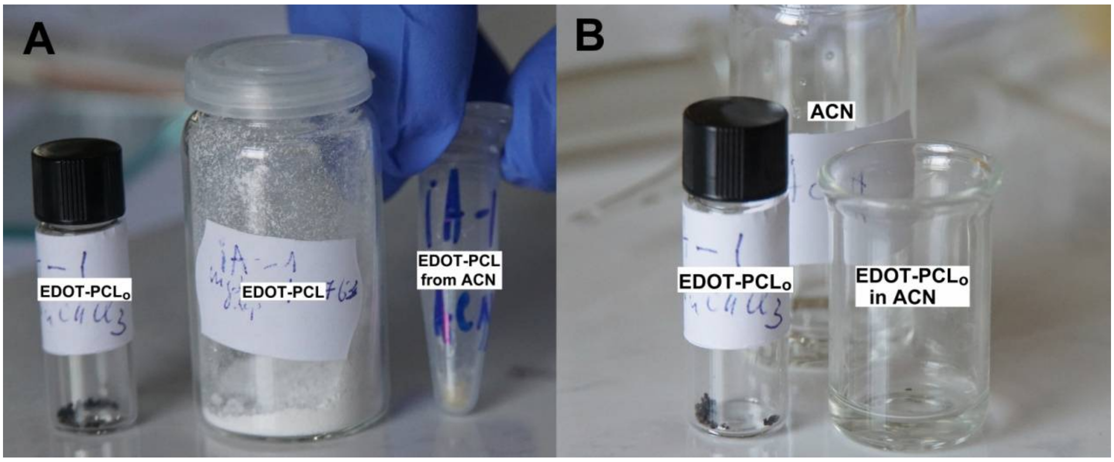

Figure S 8. (**A**)- Photo showing the bulk form of **EDOT-PCL** as resulted from the reaction as an white-grey powder (middle), of **EDOT-PCL** as resulted after evaporation of an ACN solution (right) and **EDOT-PCL** oligomerized form (**EDOT-PCL$_o$**), as resulted after evaporation of acidic $CDCl_3$ solution (left); (**B**)- Photo showing **EDOT-PCL$_o$** having dark blue colour in solid state (left) and the colourless, transparent aspect of **EDOT-PCL$_o$** ACN solution (which shows a blue colour when is solved in chloroform -see Figure 6 B in main manuscript)

References

1. Rayeroux, D.; Travelet, C.; Lapinte, V.; Borsali, R.; Robin, J.-J.; Bouilhac, C. Tunable amphiphilic graft copolymers bearing fatty chains and polyoxazoline: synthesis and self-assembly behavior in solution. *Polym. Chem.* **2017**, *8*, 4246–4263.

2. Xiang, L.; Ryu, W.; Kim, H.; Ree, M. Precise Synthesis, Properties, and Structures of Cyclic Poly(ε-caprolactone)s. *Polymers* **2018**, *10*, 577; DOI:10.3390/polym10060577.

3. Burke, J. AIC Book Paper Group Annu. Aug **1984**, *3*, 13–58. clorofrom parm solub

4. Adamska, K.; Voelkel, A.; Berlinska, A. The solubility parameter for biomedical polymers-Application of inverse gas chromatography. *J. Pharm. Biomed. Anal.* **2016**, *127*, 202–206.

5. Luo, C. J.; Stride, E.; Edirisinghe, M. Mapping the Influence of Solubility and Dielectric Constant on Electrospinning Polycaprolactone Solutions. *Macromolecules* **2012**, *45*, 4669–4680.-PCL parametru de solubilitate

6. Baysal, B.M.; Stockmayer, W. H. Direction of the dipole moment in the ester group of poly(epsilon-caprolactone). *Macromolecules*, **1994**, *27*, 7429–7432.

7. Wang, G.; Jiang, C.; Gao, C.; Wang, X.; Chen, G. Dispersion of Electrically Conductive Polymer PEDOT in Organic Solvents. Acta Polymerica Sinica **2014**, *11*, 1532–1538; DOI: 10.11777/j.issn1000-3304.2014.14094

8. Li, Y.; Fenga, Y.; Feng, W. The synthesis of poly(3,4-ethylenedioxythiophene) micro/nano-spheres by the demulsifying treatment. Synth. Met. **2012**, *162*, 781-787.
